# Sweet relief: exploring mechanisms and therapeutic approaches of sodium-glucose cotransporter-2 inhibitors in cardiovascular-kidney metabolic syndrome

**DOI:** 10.1186/s12933-026-03173-5

**Published:** 2026-04-05

**Authors:** Jiaxin Hua, Qingyuan Wang, Ye Liu, Shengwei Luo, Ao Shi, Zhiwei Yan, Jing Zhang, Wenli Gu, Lihan Zhu, Yuling Zhang, Lin Zhang, Peng Yu, Xiao Liu, Wenting Wang

**Affiliations:** 1 Jiujiang Clinical Precision Medicine Research Center, Jiujiang, China; 2 Department of Endocrinology and Metabolism, The Second Affiliated Hospital, Jiangxi Medical College, Nanchang University, Nanchang, China; 3 Centre for Behavioural & Implementation Science Interventions, Yong Loo Lin School of Medicine, National University of Singapore, Singapore, Singapore; 4 Department of Cardiology, Renji Hospital, School of Medicine, Shanghai Jiao Tong University, Shanghai 200127, China, Shanghai, China; 5 Provincial University Key Laboratory of Sport and Health Science, School of Physical Education and Sport Sciences, Fujian Normal University, Fuzhou, Fujian China; 6 Department of Anesthesiology, The Second Affiliated Hospital, Jiangxi Medical College, Nanchang University, Nanchang, China; 7 Cardiology Division, Department of Medicine, Queen Mary Hospital, The University of Hong Kong, Pok Fu Lam Rd 102. Hong Kong Island, Hong Kong, China; 8 Department of Physiology & Biomedical Sciences, Ischemic/Hypoxic Disease Institute, Seoul National University College of Medicine, Seoul, Republic of Korea; 9 Department of Cardiology, Sun Yat-sen Memorial Hospital of Sun Yat- sen University, Guangzhou, China; 10 Department of Epidemiology and Preventive Medicine, The School of Public Health and Preventive Medicine, Monash University, Clayton, Australia; 11 Cardiovascular & Metabolic Disorders Program, Duke-National University of Singapore Medical School, Singapore, Singapore; 12 Department of Anesthesiology, The Second Affiliated Hospital of Hainan Medical University, Haikou, China

**Keywords:** Cardiovascular-kidney-metabolic, SGLT-2i, Cardiorenal protection, Type 2 diabetes

## Abstract

The escalating global burden of chronic multimorbidity has necessitated a fundamental transition from organ-centric treatment models toward integrated, multisystemic management strategies. Central to this evolution is the recognition of the cardiovascular–kidney–liver-metabolic (CKM) syndrome, a clinical framework arising from the profound and bidirectional pathophysiological crosstalk between metabolic dysfunction, chronic kidney disease, and heart failure. Sodium-glucose cotransporter-2 inhibitors (SGLT-2i) have transitioned from simple glucose-lowering drug to a critical pillar of the area of cardiovascular metabolism, demonstrating profound benefits across the cardiovascular, metabolism and renal systems. These therapeutic effects are driven by a complex interplay of hemodynamic and non-hemodynamic mechanisms, including the restoration of tubuloglomerular feedback, optimization of myocardial energetics, and the attenuation of systemic inflammation and oxidative stress. This pharmacological profile aligns precisely with the multi-stage progression of CKM syndrome, offering a cohesive intervention to interrupt the vicious cycle of systemic risk. In this review, we delineated the mechanistic pathways by which SGLT-2i modulate the CKM axis and evaluate the clinical evidence supporting its important role for integrated CKM management, providing a comprehensive perspective on optimizing long-term outcomes through multisystemic targeting.

**Graphical Abstract:**

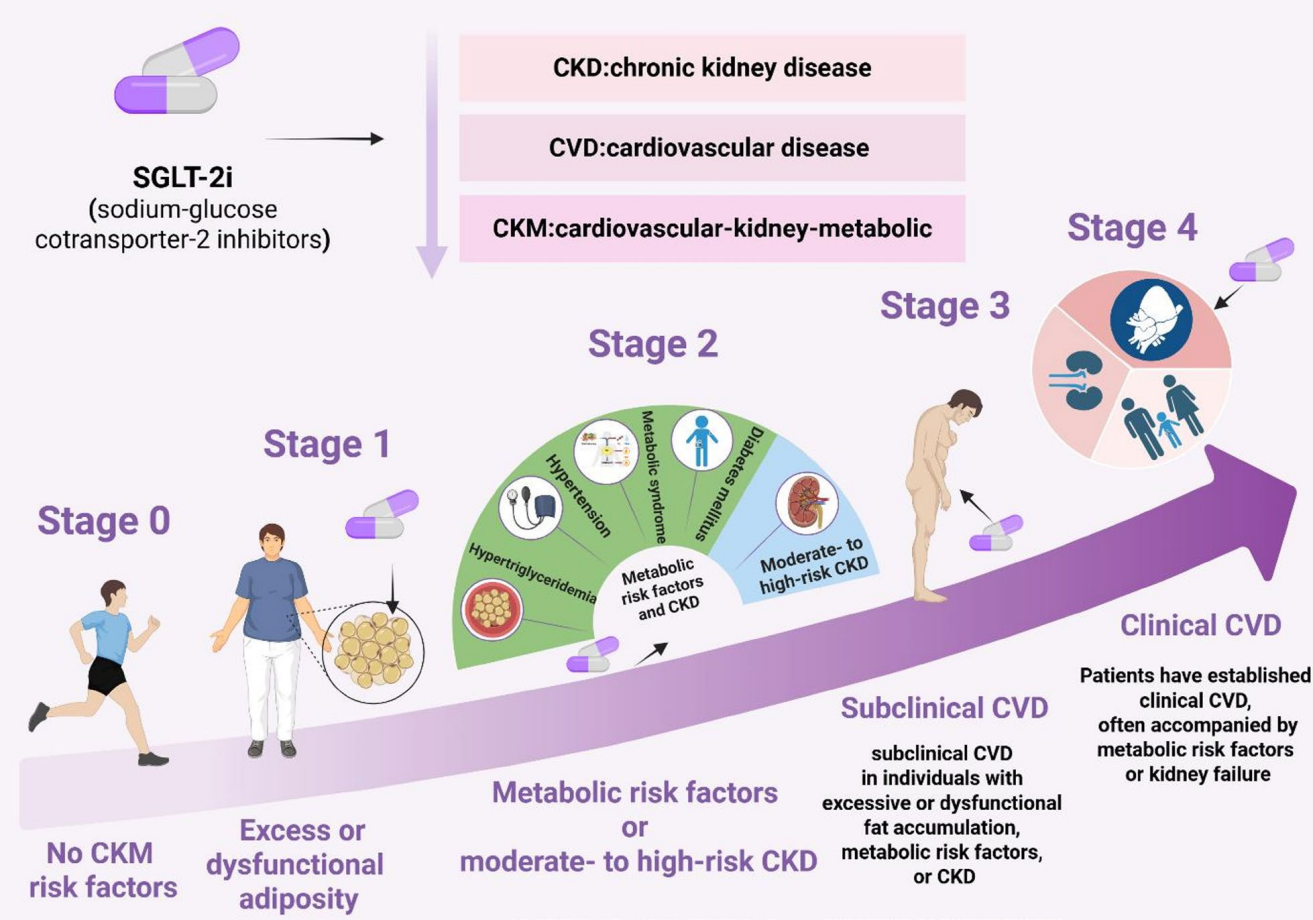

**Supplementary Information:**

The online version contains supplementary material available at 10.1186/s12933-026-03173-5.

## Introduction

Recent advances in biomedical research have uncovered a complex interplay among metabolic processes, renal function, and cardiovascular health. In 2023, the American Heart Association (AHA) introduced the term cardiovascular-kidney-metabolic (CKM) syndrome to describe this intricate network of interrelated conditions [1]. CKM syndrome is a systemic disorder characterized by pathophysiological interactions among metabolic risk factors, chronic kidney disease (CKD), and cardiovascular disease (CVD).

Current evidence suggests that CKM syndrome is a progressive disorder that originates from the accumulation of excess and dysfunctional adipose tissue [2], which triggers inflammation, oxidative stress (OS), and insulin resistance (IR) [3]. These processes contribute to the development of metabolic risk factors such as hypertension, hypertriglyceridemia, metabolic syndrome (MetS), and type 2 diabetes mellitus (T2DM), as well as CKD [4–6]. The convergence of these conditions often culminates in clinical CVD manifestations, including coronary atherosclerosis, myocardial structural and functional abnormalities, and a progressive decline in renal function [1, 7, 8].

According to the AHA, CKM syndrome is stratified into five stages, with each stage associated with specific pathophysiological features, risk factors, therapeutic options, and preventive strategies (Table 1) [9]. Despite these advancements, significant gaps remain in the clinical understanding of CKM syndrome, particularly concerning early-life prevention strategies, novel cardioprotective and nephroprotective therapies, and the comprehensive management of patients affected by both CKD and CVD [10].

**Table 1:** Pathophysiologic features, risk factors, treatment and prevention of the various stages of CKM syndrome

CKM syndrome stages	Pathophysiology	Risk factor	Treatment	Prevention	Ref.
Stage 0: No CKM risk factors	Normal BMI and waist circumference, normal blood glucose, normal blood pressure, normal blood lipids, no CKD or subclinical or clinical CVD	High-calorie food and beverage intake, sedentary lifestyle, reduced physical activity and other bad habits	Enhanced lifestyle, improved diet and increased physical activity	Biological Factors Screening and SDOH Screening Screening for Mets components is recommended every 3–5 years	[1, 9, 165, 166]
Stage 1: Excess or dysfunctional adiposity	Overweight/obesity, abdominal obesity or adipose tissue dysfunction without other metabolic risk factors	Overweight, obesity, familial obesity, etc.	Fitness using a heart-healthy diet and increased physical activity. SGLT-2i, GLP-1 RAs: semaglutide or bariatric surgery	Prevent the development of metabolic risk factors in patients with excessive or dysfunctional obesity. It is recommended to screen for Met components every 2–3 years	[1, 9, 24, 25], 169– [172]
Stage 2: Metabolic risk factors and CKD	Have metabolic risk factors (hypertriglyceridemia [≥ 135 mg/dL], hypertension, MetS+, diabetes) or CKD	Hypertension, Proteinuria are Risk Factors for CVD Hypertriglyceridemia increases ASCVD risk	Lifestyle changes, targeted drug therapy for blood pressure, blood glucose and lipids such as: statins, SGLT-2i, RAAS inhibitors. Drug combination therapy e.g. fenugreek in combination with SGLT-2i, metformin in combination with SGLT-2i.	Control metabolic risk factors to prevent progression to clinical or subclinical CVD. Annual screening for Mets components is recommended, and 1–2 years for MASLD-associated pulmonary fibrosis in patients with diabetes or pre-diabetes.	[1, 9, 26, 29, 174, 176, 179]
Stage 3: Subclinical CVD in CKM	Subclinical CVD and Risk Equivalents in CKM Subclinical ASCVD or subclinical HF. Individuals with very high risk of CKD or predicted to be at high risk of CVD are also included in stage 3 CKM.	Abnormal cardiac structure or function, cardiovascular risk factors and potential comorbidities.	Reduce ASCVD risk with drugs including aspirin therapy, PCSK9 inhibitors, statins, ACEI/ARBs and SGLT-2i.	Enhance lifestyle changes and preventive therapy based on CKM risk profile. KDIGO coronary screening is recommended, as well as annual measurement of BMI, waist circumference and Mets composition.	[1, 9, 31, 120, 191, 192, 198]
Stage 4: Clinical CVD in CKM	Clinical CVD in patients with excess/dysfunctional obesity, other CKM risk factors, or CKD. Stage 4a: absence of renal failure. stage 4b: presence of renal failure.	Coronary heart disease, heart failure, stroke, peripheral arterial disease, atrial fibrillation, and other cardiometabolic risk factors. Hypertension, obesity, CKD and dyslipidemia in CKM may all be associated with atrial fibrillation.	High-intensity statins, beta-blockers, angiotensin receptor/neprilysin inhibitors, mineralocorticoid receptor antagonists, and SGLT-2i Drug combination therapy e.g. SGLT-2 i + GLP-1 RAs Different CKM risk factors require additional treatment considerations.	Focusing throughout on the care and secondary prevention of patients with CVD combined with metabolic factors, CKD, or both. Emphasize the 8 basic frameworks of life. It is recommended that BMI and waist circumference should be measured at least annually, with the presence of obesity and abdominal obesity indicating a particular need for weight loss.	[1, 9, 99, 208, 210, 211]

CKM: cardiovascular-kidney-metabolic; BMI: body mass index; CKD: chronic kidney disease; CVD: cardiovascular disease; SDOH: social determinants of health; Met: metabolic syndrome; ASCVD: atherosclerotic cardiovascular disease; HF: heart failure; SGLT-2i: sodium-glucose cotransporter-2 inhibitors; RAAS: renin-angiotensin-aldosterone system; MASLD: metabolic dysfunction–associated steatosis liver disease; PCSK9: Proprotein Convertase Subtilisin/Kexin Type 9; ACEI: angiotensin-converting enzyme inhibitors; ARBs: Angiotensin II Receptor Blockers; KDIGO: Kidney Disease Improving Global Outcomes

Sodium-glucose cotransporter-2 inhibitors (SGLT-2i) are primarily known as a novel class of hypoglycemic agents for the clinical management of T2DM. However, accumulating evidence indicates that SGLT-2i also exerts substantial cardioprotective and nephroprotective effects [11, 12]. Large-scale clinical trials have demonstrated their efficacy in addressing multiple components of CKM syndrome [13–16]. According to the 2020 guidelines of the American Diabetes Association (ADA), SGLT-2i are recommended for patients with T2DM who have established atherosclerotic cardiovascular disease (ASCVD) or are at high risk for CVD, CKD, or heart failure (HF) [17]. These findings support the potential use of SGLT-2i as a therapeutic strategy within the context of CKM syndrome. This review will propose a novel therapeutic concept for the application of SGLT-2 inhibitors in CKM syndrome and discuss the underlying pathophysiological mechanisms that support this approach. The search strategy is shown in Supplemental Table S1.

## CKM syndrome

Cardiovascular, renal and metabolic diseases often overlap and coexist in the same patient. Diabetes is the main cause of chronic kidney disease (CKD) [18], and is associated with adverse cardiovascular outcomes. When there is renal dysfunction, the cardiovascular mortality rate further increases [19]. In a study of the prevalence and comorbidity of central renal metabolic diseases among 530,747 adult patients with type 2 diabetes in the United States, it was found that approximately 51% of the participants had three or more other CKD-related diseases [1]. A large amount of epidemiological evidence supports the bidirectional relationship between T2DM, HF and chronic kidney disease, and CKM syndrome links the three more systematically.

**Fig. 1:**
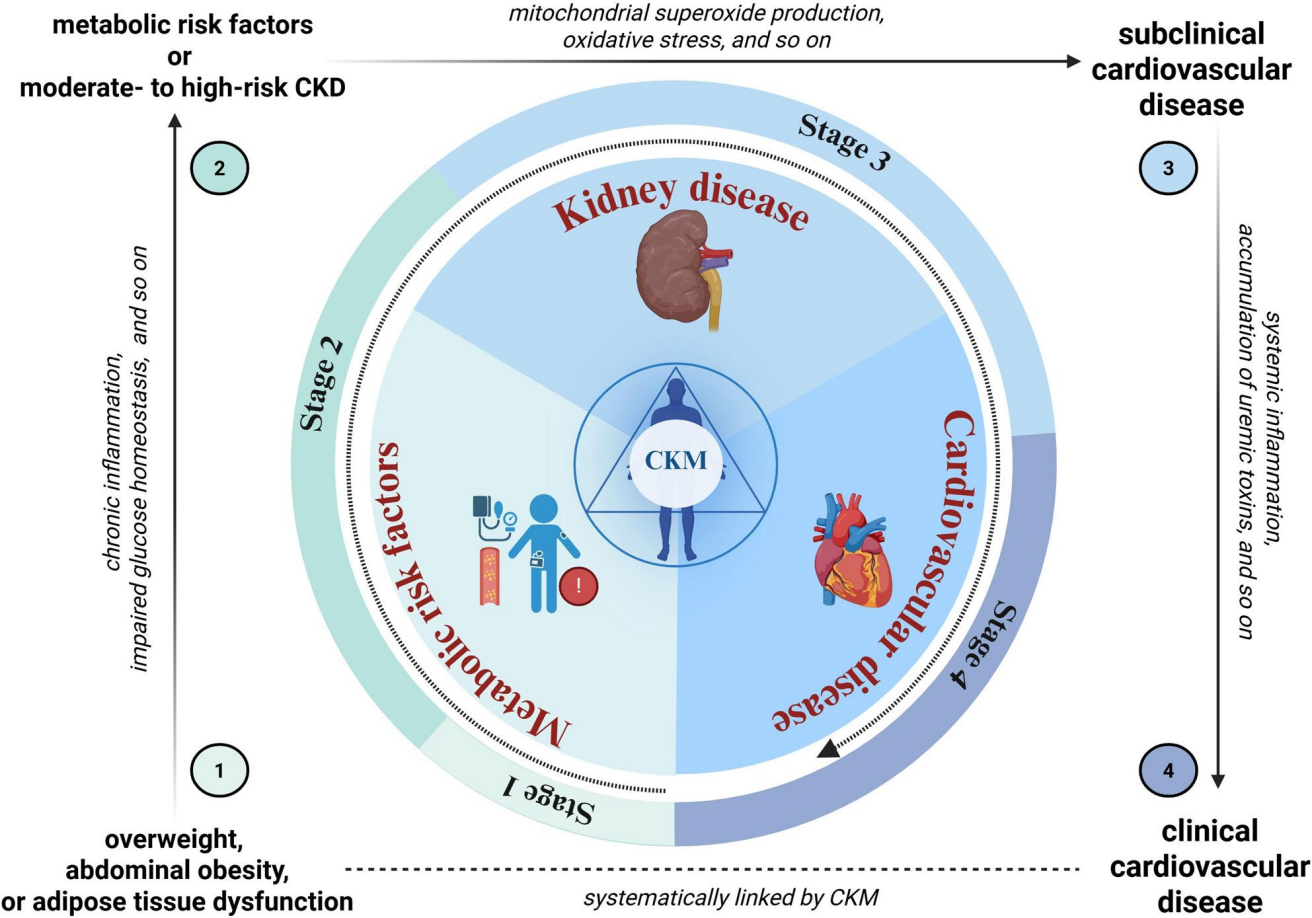
Cardiovascular-Kidney-Metabolic Syndrome. CKM syndrome is a systemic disorder marked by pathophysiological interactions among metabolic risk factors, chronic kidney disease, and the cardiovascular system. These interactions contribute to a heightened incidence of multi-organ dysfunction and a poor cardiovascular prognosis. Stage 1 typically presents with overweight, abdominal obesity, or adipose tissue dysfunction, which drives chronic inflammation and impaired glucose homeostasis. These pathophysiological changes lead to Stage 2, characterized by metabolic risk factors or moderate- to high-risk CKD. As the syndrome progresses, Stage 3 is characterized by subclinical cardiovascular disease in individuals with excessive or dysfunctional fat accumulation, metabolic risk factors, or CKD. Concurrently or subsequently, subclinical cardiovascular disease develops, advancing to Stage 4 with clinical cardiovascular disease. The central CKM axis integrates these components, emphasizing that CKM syndrome is not merely a linear cascade but a systemic network where metabolic, renal, and cardiovascular pathologies perpetuate one another. CKM, cardiovascular-kidney-metabolic; CKD, chronic kidney disease

This syndrome primarily stems from excessive and/or dysfunctional adipose tissue, which secretes pro‑inflammatory and pro‑oxidative products, leading to damage in arterial, cardiac, and renal tissues as well as reduced insulin sensitivity [20] (Fig. 1).

Individuals in CKM stage 0 have no identifiable risk factors, and the optimal strategy at this stage is lifestyle modification [9]. Those in CKM stage 1 typically present with overweight, abdominal obesity, or adipose tissue dysfunction [9]. Obesity is a significant risk factor for T2DM [21–23]. At this stage, adipose tissue dysfunction serves as the primary pathogenic driver. The key underlying mechanism involves dysfunctional adipose tissue, which promotes chronic inflammation, impaired glucose homeostasis, and defective adipogenesis, thereby triggering ectopic fat accumulation, insulin resistance (IR), and increased susceptibility to T2DM [24]. Moreover, excessive obesity predisposes individuals to vascular diseases [25].

Patients at CKM stage 2 typically exhibit metabolic risk factors—such as hypertriglyceridemia, hypertension, metabolic syndrome (MetS), or diabetes mellitus—or moderate- to high-risk CKD, or both [24]. In this phase, under hyperglycemic conditions, excess intracellular glucose flux leads to mitochondrial superoxide production and exacerbates oxidative stress (OS), which is considered a major initiator of diabetes‑induced organ damage [26]. Increased generation of reactive oxygen species (ROS) contributes to tissue injury through multiple mechanisms and activates protein kinase C (PKC) while promoting the formation of advanced glycation end‑products (AGEs) [27]. These processes directly impair cardiac, vascular, and renal tissues, as well as endothelial function [28]. Concurrently, mechanisms such as activation of the renin‑angiotensin‑aldosterone system (RAAS), sympathetic overactivity, and sodium retention promote early glomerular hyperfiltration. Sustained hyperfiltration results in progressive and irreversible damage to nephrons and a decline in estimated glomerular filtration rate (eGFR), aggravating organ dysfunction [29]. Renal tissue injury manifests as albuminuria and proteinuria, which in turn exacerbate metabolic dysregulation [30]. Endothelial dysfunction, vascular abnormalities, and inflammation are key drivers of atherosclerotic plaque formation and progression and the associated metabolic alterations may also directly affect myocardial function.

CKM stage 3 is characterized by subclinical cardiovascular disease in individuals with excessive or dysfunctional fat accumulation, metabolic risk factors, or CKD [9]. At this point, systemic inflammation, accumulation of uremic toxins, and disordered mineral‑bone metabolism exacerbate vascular calcification and myocardial fibrosis. Chronic volume overload and adverse cardiac remodeling further accelerate a vicious cycle that promotes conditions such as heart failure. Low cardiac output, reduced effective circulating volume, and excessive vasoconstrictor mediators contribute to chronic renal hypoperfusion and declining eGFR, favoring the onset and/or progression of CKD [31]. Conversely, sodium and water retention in CKD along with chronic RAAS activation worsen hypertension and increase cardiac preload and afterload. These hemodynamic disturbances, combined with the presence of CKD, form a self‑perpetuating pathological cycle [31].

Patients with stage 4 CKM syndrome generally have established clinical cardiovascular disease, often accompanied by metabolic risk factors or kidney failure [9]. The pathological mechanisms are dominated by irreversible vascular stiffness, profound neuro‑hormonal activation, and systemic multiorgan dysregulation. Clinical manifestations include myocardial infarction, stroke, sudden cardiac death, and dialysis‑dependent kidney failure, all of which substantially increase patient mortality.

## Potential mechanisms of sodium-glucose cotransporter-2 inhibitors (SGLT-2i) in the treatment of CKM syndrome

CKM syndrome represents a systemic condition involving complex interactions between metabolic risk factors, CKD, and the cardiovascular system [9]. SGLT-2i exert multifaceted therapeutic effects by preventing the development of metabolic disorders, enhancing renal function, and promoting cardiovascular health [32–34]. This section explores the potential mechanisms underlying the therapeutic effects of SGLT-2i in CKM syndrome (Table 2).

**Table 2:** Summary of preclinical studies titled potential mechanisms of SGLT-2i in the treatment of CKM syndrome

Model or diseases	Treatment	Mechanism	Ref.
Diabetic fatty rats model	CANA	Increasing urinary glucose excretion and decreasing blood glucose	[38]
Obese T2DM mouse model	DAPA	Restoring endogenous β-cell function and insulin secretion	[41]
Obese insulin-resistant mouse model	DAPA	Ameliorating obesity-induced hepatic gluconeogenesis	[42]
Mouse model with insulin receptor and insulin-like growth factor-1 receptor inhibition	LUSEO	Supporting β-cell function	[43]
Otsuka Long-Evans Tokushima Fatty rat model	DAPA	Controlling hypertension by reducing urinary levels of angiotensin II and angiotensinogen	[47]
Non-diabetic CKD rat model	LUSEO	Controlling hypertension by attenuating salt sensitivity and sympathetic tone	[50]
High-fat diet-fed ApoE(-/-) mouse model	EMPA	Inhibiting the development of hypertriglyceridemia by reducing total cholesterol, serum TG levels, and lipogenic enzyme expression	[55]
Diabetic mouse model expressing human cholesteryl ester transfer protein and apolipoprotein B100	CANA	Inhibiting the development of hypertriglyceridemia by increasing low-density lipoprotein cholesterol, decreasing plasma TG levels, reducing postprandial lipemia and accelerating clearance of radiolabeled very-low-density lipoprotein	[57]
Zucker diabetic fatty rat model	CANA	Inhibiting the development of hypertriglyceridemia by improving IR status	[58]
Zucker diabetic fatty rat model	EMPA	Attenuating cardiac metabolic disorders and inflammation	[63]
Streptozotocin-induced diabetic rat model	EMPA	Improving metabolic status by reversing the pro-inflammatory phenotype and glucotoxicity	[64]
High-fat diet-induced obese mouse model	EMPA	Attenuating obesity-induced inflammation and IR	[68]
High-fat-diet-induced IR dog model	DAPA	Enhancing adipose tissue function in regulating energy homeostasis	[70]
KK Cg-Ay/J mouse model	EMPA	Improving metabolic health by promoting mitochondrial biogenesis and fusion, improving mitochondrial function and integrity, and inducing adipocyte browning	[71]
High-fat diet-induced NAFLD mice model	EMPA	Mitigating the progression of NAFLD by downregulating hepatic lipogenic gene expression	[77]
Type 2 diabetic mice model	IPRA	Reducing serum and hepatic inflammatory cytokines	[78]
Acetaminophen-induced hepatic injury mice model	CANA	Alleviating hepatic injury by its anti-inflammatory and antioxidant effects	[79]
A mice model of T2DM with NAFLD	EMPA	Alleviating liver injury by modulating the interleukin-17/interleukin-23 axis	[80]
NASH model	IPRA	Alleviating NASH by reducing apoptotic cell numbers and decreasing fibrotic areas	[81]
NAFLD mice model associated with fibrosis	EMPA	Alleviating NAFLD-associated hepatic fibrosis by downregulating microRNA-34a-5p and upregulating GREM2	[82]
Type 1 diabetic Akita mouse model	EMPA	Attenuating glomerular hypertrophy and downregulating molecular markers associated with kidney growth, inflammation, and gluconeogenesis	[106]
Non-diabetic mice model with early uncomplicated hyperglycemia	DAPA	Reducing early proximal tubule glucotoxicity and extensive downregulation of apical uptake transport mechanisms for sodium, glucose, urate, purine bases and amino acids	[107]
Genetic hypertriglyceridemic rat model	EMPA	Protecting kidney by reducing lipoperoxidation products and enhancing the activity of superoxide dismutase, glutathione-dependent enzymes, and glutathione peroxidase	[119]
High-glucose-induced oxidative stress model of human renal proximal tubular epithelial cells	EMPA	Treating kidney inflammatory response/fibrosis and nephropathy under chronic hyperglycemic conditions by inhibiting RECK expression	[120]
Human proximal tubule cells	EMPA	Inhibiting inflammatory response genes	[121]
Monocrotaline-induced pulmonary arterial hypertension rat model	CANA	Attenuating pulmonary artery and right ventricular remodeling and dysfunction in pulmonary arterial hypertension	[123]
Transverse aortic constriction-induced left ventricular hypertrophy mouse model	EMPA	Attenuating left ventricular remodeling and improving cardiac function	[124]
Db/db mouse model of severe diabetes	EMPA	Improving left ventricular diastolic function	[132]
Non-diabetic porcine heart failure model	EMPA	Improving diastolic function by reducing interstitial myocardial fibrosis, enhancing titin phosphorylation, alleviating both left ventricle and cardiomyocyte stiffness and mitigating histological and molecular remodeling	[133]
Western diet-fed mouse model of ischemia/reperfusion	EMPA	Reducing infarct size and improving myocardial function by activating STAT3 signaling pathway	[138]
Non-diabetic porcine model of ischemia/reperfusion	EMPA	Improving myocardial salvage and preserving cardiac function by increasing circulating ketone body levels	[142]
Transverse aortic constriction-induced HF mouse model	EMPA	Attenuating left ventricular remodeling and pressure-overload HF	[145]
Diabetic cardiomyopathy mouse model	EMPA	Alleviating diabetes-related myocardial injury by reactivating the autophagy activity that is typically suppressed in diabetes	[146]
Diabetic rat model	EMPA	Improving cardiac function by alleviating myocardial oxidative stress and fibrosis through AMPK signaling pathways in the atrium	[126]
Diabetic mouse model	EMPA	Alleviating myocardial oxidative stress and fibrosis by inhibiting the Transforming Growth Factor β/Smad signaling pathway and activating the Nuclear factor erythroid 2-related factor 2/Antioxidant Response Element signaling pathway	[148]
HF rodent models	EMPA	Decreasing cardiac inflammation and maintaining cardiac function	[127]
Human coronary arterial endothelial cells subjected to cyclic stretch	EMPA	Ameliorating endothelial damage by decreasing the production of reactive oxygen species	[153]
Obese ZSF1 rat model	EMPA	Ameliorating endothelial dysfunction by reducing the expression of endothelial senescence markers (p53, p21, p16)	[154]
T2DM mice model	DAPA	Ameliorating endothelial dysfunction by increasing endothelial nitric oxide synthase and nitric oxide	[155]
Streptozotocin-induced diabetic rat model	EMPA	Inducing coronary vasodilation in diabetes	[157]
Prediabetic ob/ob^(−/−)^ mouse model	EMPA	Enhancing coronary flow velocity reserve and fractional area change	[158]

CANA: canagliflozin; DAPA: dapagliflozin; EMPA: empagliflozin; LUSEO: luseogliflozin; IPRA: ipragliflozin; IR: insulin resistance; CKD: chronic kidney disease; NAFLD: nonalcoholic fatty liver disease; T2DM: type 2 diabetes; TG: triglyceride; NASH: nonalcoholic steatohepatitis; AMPK: adenosine monophosphate-activated protein kinase; RECK: reversion inducing cysteine-rich protein with Kazal Motifs; HF: heart failure

### Attenuation of metabolic disease progression

Metabolic risk factors—such as hypertriglyceridemia, hypertension, diabetes mellitus (DM), dyslipidemia, and metabolic syndrome—play a central role in the pathogenesis of CKD and contribute significantly to adverse cardiovascular and renal outcomes. These factors represent critical intervention targets in the management of CKM syndrome [9]. SGLT-2i offer a promising strategy to prevent the onset of metabolic disorders, particularly DM, thereby impeding the progression of associated complications and improving patient prognosis.

**Fig. 2:**
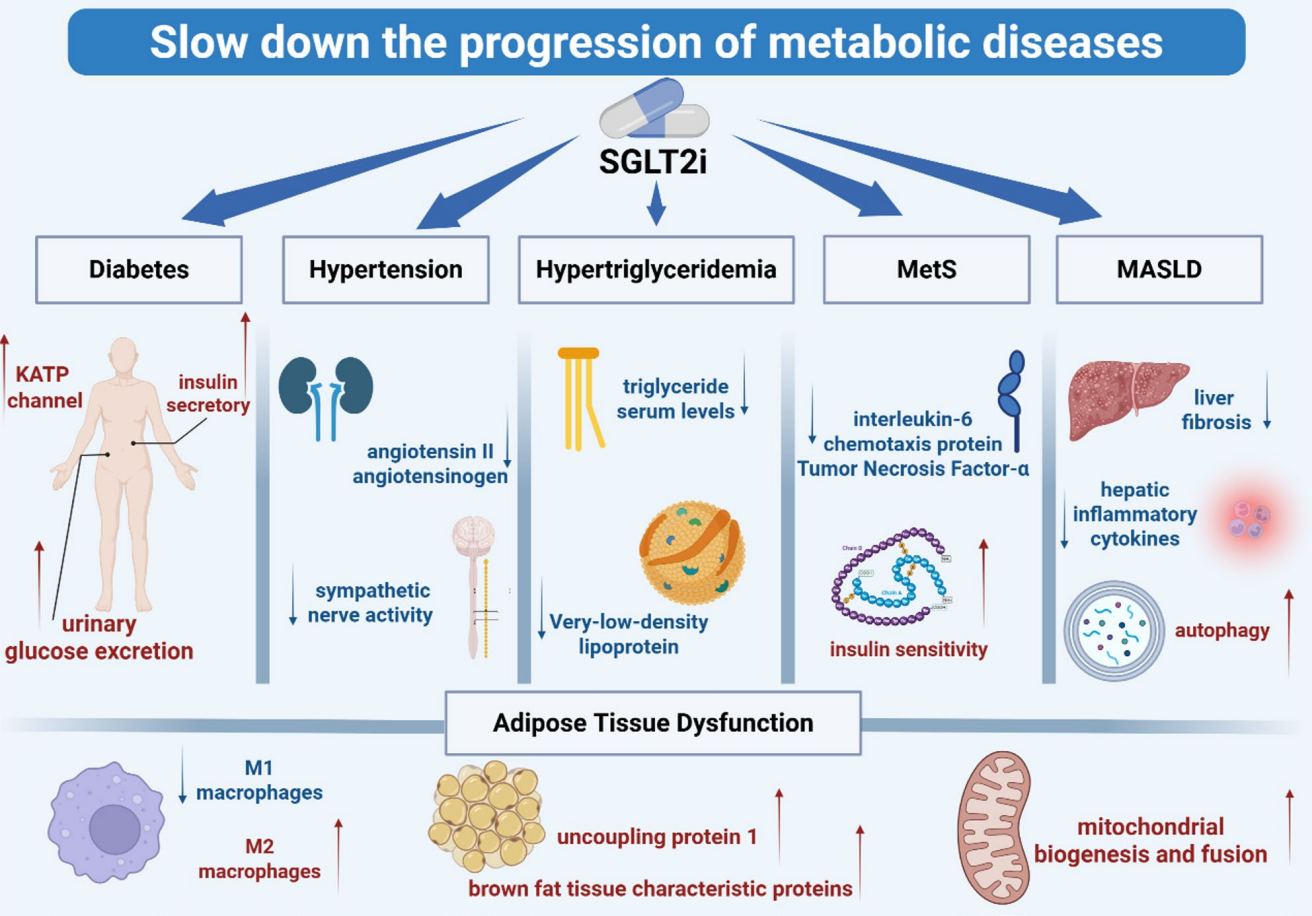
The mechanism by which SGLT-2i mitigates the progression of metabolic risk factors. SGLT-2i ameliorate diabetes by boosting urinary glucose excretion, activating KATP channels, and enhancing insulin secretion, thereby lowering blood glucose. SGLT-2i also modulate hypertension by curbing renal angiotensinogen activity, decreasing angiotensin II production and dampening sympathetic nerve activity. In hypertriglyceridemia, SGLT-2i reduce serum triglyceride levels and inhibit very-low-density lipoprotein generation. For MetS, SGLT-2i regulate attenuate adipose tissue inflammation and heighten insulin sensitivity. In MASLD, SGLT-2i cut down hepatic inflammatory cytokine release and ease liver fibrosis. Moreover, SGLT-2i adjust adipose tissue function by promoting M2 macrophage polarization, up-regulating brown fat-specific like proteins uncoupling protein 1, bolstering mitochondrial biogenesis and fusion, and inducing adipose tissue autophagy, all of which improve adipose tissue function. SGLT-2i: sodium-glucose cotransporter-2 inhibitors; KATP: ATP-sensitive potassium channel; MetS: metabolic syndrome; MASLD: metabolic dysfunction-associated fatty liver disease

Moreover, given the potential influence of SGLT-2i on metabolic dysfunction-associated steatotic liver disease (MASLD), further investigation into their therapeutic role in MASLD is warranted (Fig. 2).

#### Diabetes

Sodium-glucose co-transporter 2 (SGLT-2) is a key transporter responsible for the majority of glucose reabsorption in the proximal tubules of the kidneys [35]. Inhibition of SGLT-2 reduces the renal capacity for glucose reabsorption, constituting the fundamental mechanism underlying the therapeutic use of SGLT-2i in diabetes management [36]. Although SGLT-2 is responsible for 80–90% of renal glucose reabsorption under normal physiological conditions, due to the compensatory effect of Sodium-glucose cotransporter 1 (SGLT-1), even the maximum dose of SGLT-2i produce only about 50–60 g of glucosuria per 24 h (about 30–50% of the filtered glucose load) [37]. This partial inhibition characteristic endows SGLT-2i with a certain degree of safety in the treatment of diabetes, but also restricts further enhancement of their glucose-lowering effect. In diabetic fatty rats, treatment with canagliflozin (CANA) for four weeks resulted in increased urinary glucose excretion and decreased blood glucose levels, thereby improving glycemic control in a rodent model of T2DM [38]. However, this hypoglycemic effect is not sustained indefinitely, as compensatory mechanisms may lead to increased endogenous glucose production. These mechanisms include alterations in glycemic levels, reduced insulin-to-glucagon ratios in the hepatic portal circulation, and rapid elevations in circulating free fatty acid levels [39]. Furthermore, SGLT-2i promote glucagon secretion and hepatic gluconeogenesis through the activation of ATP-sensitive potassium (KATP) channels, thus contributing to the maintenance of normoglycemia [39]. Glycemic control not only mitigates metabolic syndrome but also reduces cardiac and renal burdens, underscoring its importance in the management of CKM syndrome.

Impaired insulin secretion by pancreatic β-cells and insulin resistance in peripheral tissues are central to the pathophysiology of T2DM [40]. The therapeutic effects of SGLT-2i may be partially attributed to the restoration of endogenous β-cell function and insulin secretory capacity. In a murine model of obesity-induced T2DM characterized by β-cell failure, DAPA treatment for four weeks increased the number of insulin granules and indirectly enhanced β-cell function and insulin secretion [41]. Similarly, in obese insulin-resistant mice, four weeks of SGLT-2i treatment ameliorated obesity-induced hepatic gluconeogenesis by restoring hepatic insulin signaling [42]. In a mouse model with inhibition of insulin and insulin-like growth factor-1 (IGF-1) receptors, one week of luseogliflozin treatment significantly increased β-cell proliferation and upregulated genes in the forkhead box M1/polo-like kinase 1/centromere protein A pathway, suggesting a humoral mechanism that supports β-cell function independently of insulin and IGF-1 receptor signaling [43].

#### Hypertension

Hypertension arises through various mechanisms, including increased sodium absorption, dysregulation of the RAAS, and enhanced sympathetic nervous system activity, all of which contribute to elevated total peripheral resistance and increased afterload [44].

RAAS is a crucial regulatory system involved in controlling plasma sodium concentration, arterial pressure, and extracellular fluid volume [45]. As a result, RAAS inhibitors have become central to hypertension treatment strategies [46]. In an Otsuka Long-Evans Tokushima Fatty rat model, 12 weeks of DAPA treatment significantly reduced urinary levels of angiotensin II and angiotensinogen, suggesting its potential in blood pressure regulation [47]. Additional studies have highlighted the roles of sympathetic nervous system activation and sodium intake in blood pressure modulation [48, 49]. In a non-diabetic CKD rat model induced by adenine, five days of luseogliflozin treatment decreased salt-induced elevations in mean arterial pressure and sympathetic nerve activity, indicating that luseogliflozin may reduce blood pressure by attenuating salt sensitivity and sympathetic tone [50]. Impaired renal function results in sodium and fluid retention, exacerbating hypertension and demonstrating the kidneys’ essential role in blood pressure homeostasis [51]. SGLT-2i provide renal protection via multiple mechanisms, thereby aiding in blood pressure control [52].

#### Hypertriglyceridemia

Hypertriglyceridemia is a core feature of metabolic syndrome and a significant risk factor for coronary artery atherosclerosis [53]. The pathogenesis of hypertriglyceridemia involves an imbalance in triglyceride (TG) synthesis, transport, and degradation [54].

In a high-fat diet-fed mouse model, five weeks of EMPA treatment reduced total cholesterol, serum TG levels, and lipogenic enzyme expression, indicating its potential to inhibit hypertriglyceridemia development [55]. Very-low-density lipoprotein (VLDL) serves as the primary TG carrier in circulation, and increased synthesis or impaired clearance of VLDL results in elevated plasma TG concentrations [56]. In a diabetic mouse model expressing human cholesteryl ester transfer protein (CETP) and apolipoprotein B100 (ApoB100), four weeks of CANA treatment led to increased low-density lipoprotein cholesterol, decreased plasma TG levels, reduced postprandial lipemia, and accelerated clearance of radiolabeled VLDL, contributing to the prevention of hypertriglyceridemia [57]. In IR states, increased VLDL secretion, elevated TG levels, and hepatic steatosis are commonly observed [3]. In a Zucker diabetic fatty rat model, seven days of CANA treatment improved skeletal muscle insulin resistance [58], suggesting that SGLT-2i may ameliorate hypertriglyceridemia by improving insulin sensitivity [58]. Additionally, secondary hypertriglyceridemia—associated with factors such as obesity, metabolic syndrome, and diabetes—may also be prevented by SGLT-2i [59].

#### Metabolic syndrome

MetS, as defined by the World Health Organization, is a pathological condition characterized by abdominal obesity, IR, hypertension, and hyperlipidemia [60]. Among these components, IR is considered a core pathological mechanism of MetS [61]. SGLT-2i may mitigate the progression of MetS by improving IR [58]. Inflammatory signaling pathways can directly or indirectly disrupt insulin action, thereby contributing to the development and progression of metabolic disorders [62]. In a Zucker diabetic fatty rat model, treatment with EMPA for six weeks significantly reduced cardiac mRNA expression levels of pro-inflammatory cytokines, including interleukin-6, chemotactic protein, tumor necrosis factor-α, and monocyte chemoattractant protein-1, thereby attenuating cardiac metabolic dysfunction and inflammation [63]. In a streptozotocin-induced diabetic rat model, EMPA administration for seven weeks significantly reversed glucotoxicity and pro-inflammatory phenotypes by modulating advanced glycation end product/receptor for advanced glycation end product (AGE/RAGE) signaling, contributing to metabolic improvement [64]. These findings suggest that SGLT-2i may exert therapeutic effects against MetS by modulating inflammatory pathways. Chronic inflammation is an important pathophysiological mechanism of obesity [65]. SGLT-2i may reduce inflammation and improve IR, thereby enhancing insulin’s action in promoting lipolysis and achieving weight loss. In addition, by reducing glucose reabsorption in the proximal tubules, SGLT-2i increase urinary glucose excretion and reduce blood glucose levels, which in turn promotes weight loss [66]. This is one of the most important mechanisms for their weight-loss effect.

#### Adipose tissue dysfunction

Obesity-induced adipose tissue dysfunction and inflammation lead to lipotoxicity and systemic inflammation, which collectively promote IR [67]. These interrelated factors exacerbate one another, thereby accelerating the development of metabolic disorders and underscoring the importance of obesity prevention in the early management of CKM syndrome. In a high-fat diet-induced obese mouse model, treatment with EMPA for 16 weeks significantly reduced plasma tumor necrosis factor-α levels and decreased M1 macrophage infiltration while promoting an anti-inflammatory M2 macrophage phenotype in both white adipose tissue and liver. These changes contributed to the attenuation of obesity-induced inflammation and IR [68], suggesting that SGLT-2i may prevent the progression of MetS by disrupting the pathophysiological transition from obesity to IR.

White and thermogenic adipocytes play essential roles in systemic energy homeostasis. Dysregulation of their associated pathways has been strongly linked to metabolic disorders and adipose tissue dysfunction [69]. In a high-fat diet-induced insulin-resistant dog model, DAPA treatment for six weeks was associated with normalized fat mass, increased expression of genes related to beiging, lipolysis, and adiponectin secretion, elevated AMP-activated protein kinase (AMPK) activity, and decreased levels of pro-inflammatory cytokines and ceramide synthases in both subcutaneous and visceral adipose tissue. These findings suggest that DAPA may improve adipose tissue function and energy balance regulation [70]. In the KK Cg-Ay/J mouse model, eight-week EMPA treatment resulted in reduced body weight and upregulation of uncoupling protein 1 and other brown adipose tissue-specific markers in epididymal and perirenal white adipose tissue [71]. EMPA also promoted mitochondrial biogenesis and fusion, improved mitochondrial function and integrity, and induced adipocyte browning via AMPK pathway activation, supporting its potential to enhance mitochondrial function and metabolic health [71].

#### Metabolic-associated steatotic liver disease

MASLD, formerly known as non-alcoholic fatty liver disease (NAFLD), is defined as steatotic liver disease (SLD) in the presence of one or more cardiometabolic risk factor(s) and the absence of harmful alcohol intake [72, 73]. It has become the most prevalent chronic liver disease worldwide, frequently comorbid with T2DM, hypertension, obesity, and cardiovascular disease (CVD) [74, 75]. According to the European Association for the Study of the Liver-European Association for the Study of Diabetes-European Association for the Study of Obesity (EASL-EASD-EASO) Clinical Practice Guidelines, despite the current lack of controlled clinical trials with liver histological endpoints, SGLT-2i still show certain therapeutic potential in MASLD, including a moderate reduction in liver lipid content and alanine aminotransferase (ALT) levels, which may be related to their mechanisms of action such as weight loss and improved fat metabolism [72]. Moreover, the guidelines indicate that the use of SGLT-2i is safe in MASLD. It is strongly recommended that patients with MASLD who also have type 2 diabetes, heart failure, and chronic kidney disease should continue to use these drugs to treat their respective indications [72]. Therefore, SGLT-2i has been explored as a potential therapeutic agent for MASLD, and this section will discuss the potential mechanisms of SGLT-2i in treating MASLD from the aspects of delaying the progression of metabolic syndrome, alleviating liver inflammation, and inhibiting liver fibrosis.

Slowing the progression of MetS may represent a primary mechanism by which SGLT-2i exert beneficial effects in MASLD. Hepatic fatty acid accumulation, both a cause and a consequence of NAFLD, is strongly linked to MetS [76]. In five-week-old ApoE^(−/−)^ mice fed a high-fat diet, five weeks of EMPA treatment significantly reduced fasting glucose, total cholesterol, and serum TG levels, thereby mitigating NAFLD progression [55]. In a transgenic mouse model expressing human CETP and ApoB100, SGLT-2i increased post-heparin plasma lipoprotein lipase (LpL) activity and reduced postprandial lipemia [57]. The ability to improve hyperlipidemia may constitute a crucial mechanism underlying the therapeutic potential of SGLT-2i in MASLD. In a high-fat diet-induced NAFLD mouse model, five weeks of EMPA treatment downregulated hepatic lipogenic gene expression, thus attenuating NAFLD progression [77]. Therefore, inhibition of hepatic lipogenesis may be a key mechanism by which SGLT-2i benefit MASLD.

Anti-inflammatory effects also play a major role. In a T2DM mouse model, ipragliflozin treatment reduced serum and hepatic levels of inflammatory cytokines, including interleukin-6, tumor necrosis factor-α, monocyte chemotactic protein-1, and C-reactive protein [78]. In a mouse model of acetaminophen-induced hepatic injury, five days of CANA treatment alleviated hepatic injury by modulating the p-AMPK-α/STAT-3/SOCS-3 and GSK3β/Fyn-kinase/Nrf2 signaling pathways, exerting anti-inflammatory and antioxidant effects [79]. Similarly, in a T2DM mouse model with NAFLD, EMPA treatment alleviated liver injury by modulating the interleukin-17/interleukin-23 axis, highlighting its anti-inflammatory properties [80].

#### Inhibition of liver fibrosis and promotion of autophagy

The inhibition of liver fibrosis progression and the promotion of autophagy both contribute to the hepatoprotective effects of SGLT-2i. In a nonalcoholic steatohepatitis (NASH) model, treatment with ipragliflozin for eight weeks alleviated NASH by reducing apoptotic cell numbers and decreasing fibrotic areas [81]. In a NAFLD mouse model associated with fibrosis, EMPA treatment mitigated NAFLD-related hepatic fibrosis by downregulating miR-34a-5p and upregulating GREM2, thereby inhibiting the transforming growth factor-β signaling pathway in hepatic stellate cells, which are key mediators of fibrosis [82, 83]. In ApoE^(−/−)^ mice subjected to a high-fat diet, EMPA treatment for five weeks attenuated NAFLD progression by promoting autophagy, reducing endoplasmic reticulum stress, and inhibiting hepatocyte apoptosis [55]. In a T2DM mouse model with NAFLD, EMPA enhanced autophagy in hepatic macrophages via activation of the AMPK/mammalian target of rapamycin (mTOR) signaling pathway [80].

Current clinical studies have demonstrated the beneficial effects of SGLT-2i on MASLD. In a nationwide cohort study utilizing the Korean National Health Insurance Claims Database (2014–2022), SGLT-2i use was associated with a reduced risk of hepatic decompensation events compared to thiazolidinediones (hazard ratio [HR] 0.77, 95% confidence interval [CI] 0.72–0.82) and a comparable risk to that of glucagon-like peptide-1 receptor agonists (GLP-1 RAs; HR 0.93, 95% CI 0.76–1.14) in patients with MASLD [84]. In a 48-week randomized, open-label, active-controlled trial involving biopsy-proven NAFLD patients with T2DM (*N* = 40), the tofogliflozin group (20 mg daily) exhibited significant improvements in fibrosis (*P* = 0.001), steatosis (*P* = 0.001), hepatocellular ballooning (*P* = 0.002), and lobular inflammation (*P* = 0.003) compared to baseline. In contrast, the glimepiride group showed significant improvement only in hepatocellular ballooning (*P* = 0.025), suggesting superior histological benefits of tofogliflozin in NAFLD patients with T2DM [85]. A systematic review encompassing 117 controlled trials involving patients with T2DM and MASLD/metabolic dysfunction-associated steatohepatitis (MASH) (*N* = 64,708) demonstrated robust evidence supporting SGLT-2i in regressing liver fibrosis and MASH, indicating their potential as preferred pharmacological agents in this population [86]. Similarly, a systematic review and meta-analysis of 11 randomized controlled trials (RCTs) involving NAFLD patients (*N* = 805) found that SGLT-2i significantly improved liver enzyme levels, body weight, body mass index, TG, high-density lipoprotein cholesterol, and glucose homeostasis, reinforcing their promise as therapeutic agents for NAFLD, particularly in patients with concomitant T2DM [87].

### Potential renal protective mechanisms

**Fig. 3:**
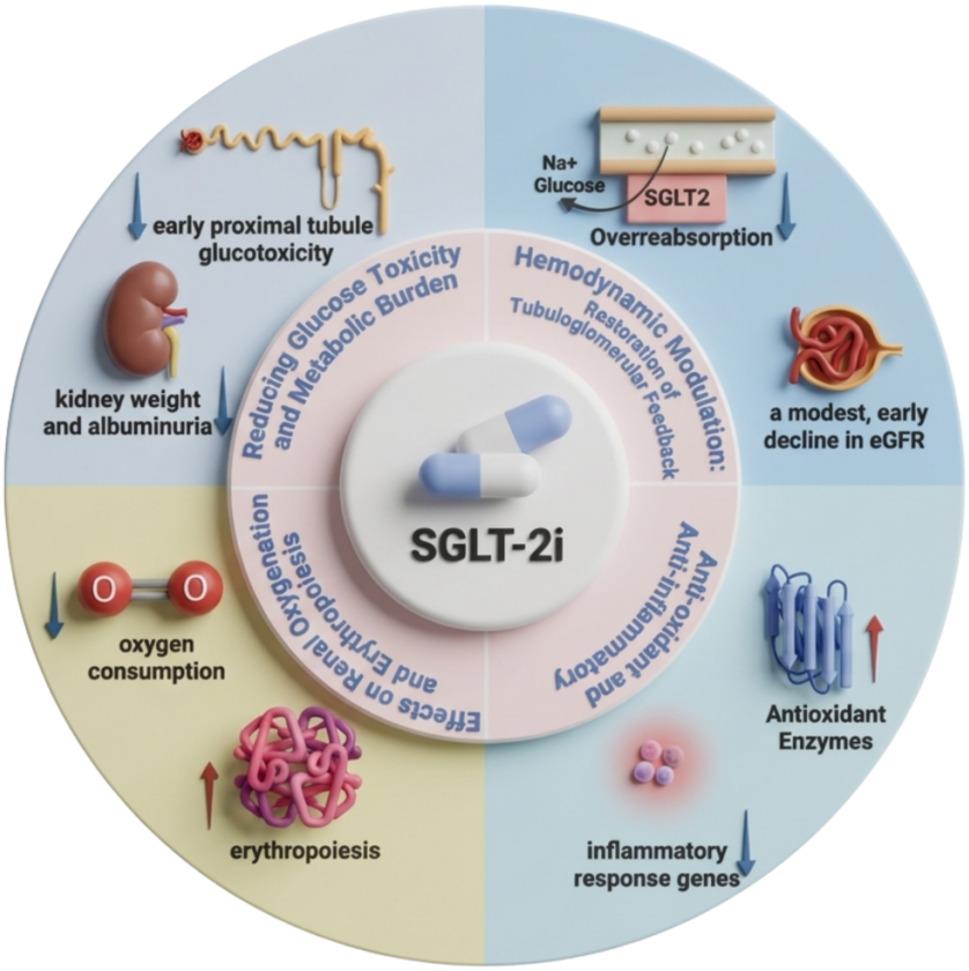
The mechanism of renal protection for SGLT-2i. Hyperglycemia upregulates SGLT2 expression in the proximal tubule, leading to overreabsorption of Na + and glucose. This disrupts tubuloglomerular feedback, causing hyperfiltration and imposing glucose toxicity and metabolic burden on the kidney. SGLT-2i interrupt this pathophysiological cascade by inhibiting SGLT2-mediated reabsorption, thereby restoring tubuloglomerular feedback and inducing a modest, early decline in eGFR. Specifically, SGLT-2i reduce early proximal tubule glucotoxicity and attenuate the increases in kidney weight and albuminuria. SGLT-2i improve renal oxygenation by decreasing oxygen consumption and enhancing erythropoiesis. Additionally, SGLT-2i exerts anti-inflammatory and anti-oxidant effects by downregulating inflammatory response genes and upregulating antioxidant enzymes. SGLT2: sodium glucose co-transporter2; SGLT-2i: sodium-glucose cotransporter-2 inhibitors; eGFR: estimated glomerular filtration rate

Renal protection is a critical component in the management of CKM syndrome. SGLT-2i exert their renal protective effects through a variety of mechanisms, including hemodynamic modulation via restoration of tubuloglomerular feedback (TGF) and reduction of intraglomerular pressure, metabolic relief through attenuation of glucose toxicity and tubular workload, and cellular protection via improved oxygenation, antioxidant defenses, and anti-inflammatory effects [88–90] (Fig. 3). Notably, the renal hemodynamic changes resulting from SGLT-2i, particularly the reduction in blood pressure and modest reduction in plasma volume, extend beyond the kidney to alleviate cardiac preload and afterload, thereby contributing to the improvement of cardiac function and the reduction in HF [88].

#### Hemodynamic modulation: restoration of tubuloglomerular feedback

TGF is an important feedback mechanism in the kidney that regulates afferent arteriolar resistance based on NaCl concentration sensed by the macula densa [91]. Under persistent hyperglycemia, the renal proximal tubule enhances glucose and sodium reabsorption via upregulated SGLT-2 transporters, reducing solute delivery to the macula densa, which inhibits TGF and adenosine-mediated afferent arteriolar vasoconstriction, thereby augmenting glomerular hyperfiltration [92]. Pathological hyperfiltration (e.g., in diabetes, obesity, or hypertension), if left untreated, is usually followed by a rapid decline in glomerular filtration rate due to progressive nephron loss, ultimately leading to chronic kidney disease and end-stage renal disease [93].

SGLT-2i counteract this pathophysiology by decreasing the reabsorption of glucose and sodium in the proximal tubule, thereby reducing intraglomerular pressure through restoration of TGF [94]. This hemodynamic change by SGLT-2i leads to a modest, early decline in eGFR (∼3 to 4 ml/min/1.73 m^2^), often reflecting a beneficial reduction in hyperfiltration rather than true kidney injury [95, 96]. After this initial dip, eGFR subsequently tends to return toward baseline and remains stable over time, ultimately resulting in long-term renoprotection and reduced albuminuria [95, 97]. The impact of SGLT2 inhibition on eGFR is consistent in patients with and without CKD, in those with established CVD [97]. This mechanism is strongly implicated in the prevention of HF onset and worsening in patients with T2DM, in both diabetic and non diabetic patients with HF irrespective of left ventricular ejection fraction phenotype [98–100], and in both diabetic and non diabetic patients with and without CKD [14, 98, 101, 102]. Thus, the restoration of TGF and the resultant hemodynamic improvements represent a unified mechanism contributing to the long-term attenuation of renal function decline and delay of CKD progression, as well as linking renal protection to cardiovascular benefits, thereby establishing SGLT-2i as integral therapy for CKM.

#### Reduction of glucose toxicity and renal metabolic burden

Beyond these hemodynamic effects, SGLT-2i confer protection by modulating tubular cell metabolism and reducing glucose toxicity. In murine models deficient in SGLT-2, studies have established the transporter’s central role in the early proximal renal tubules, mediating the majority of glucose reabsorption in the kidney [103]. When the filtered glucose load surpasses the reabsorptive capacity of these transporters, glucosuria occurs [104]. Renal biopsy specimens from patients with diabetic nephropathy have shown elevated SGLT-2 mRNA and protein expression [105], suggesting that these patients may increase sodium and glucose reuptake via upregulation of SGLT-2. SGLT-2i effectively inhibit this pathological reabsorption, thereby reducing glucose toxicity and metabolic overload in the kidney. In a type 1 diabetic Akita mouse model, EMPA treatment for 15 weeks significantly reduced blood glucose levels and mitigated the hyperglycemia-related increases in kidney weight and albuminuria. Additionally, treatment attenuated glomerular hypertrophy and downregulated molecular markers associated with kidney growth, inflammation, and gluconeogenesis, thereby contributing to renal protection [106]. In a non-diabetic hyperglycemic mouse model, DAPA treatment for one week reduced early proximal tubule glucotoxicity and led to extensive downregulation of apical transporters for sodium, glucose, urate, purine bases, and amino acids, indicating a metabolic mechanism underlying its nephroprotective effect [107].

#### Effects on renal oxygenation and erythropoiesis

Renal oxygenation is a complex physiological process that is influenced by a variety of factors, including the pre- and post-glomerular arterial-venous O_2_ shunting, tubulovascular cross-talk, the differential control of regional kidney blood flow, the TGF mechanism and so on [108]. Sodium reabsorption is the major determinant of renal oxygen consumption [109]. As the primary mediator of glucose and sodium cotransport in the early proximal tubule (S1/S2 segments), SGLT-2 is linked with renal oxygen consumption [110]. Consequently, SGLT-2i may reduce oxygen consumption in the renal cortex by decreasing transport workload. However, this upstream blockade redistributes tubular workload, shifting sodium reabsorption to downstream nephron segments (particularly the S3 segment and medullary thick ascending limb), which may contribute to restoring the oxygen-sensitivity of erythropoietin producing cells and recovery of erythropoietin secretion [32, 111, 112].

In the renal cortex, SGLT-2i improve oxygenation primarily by reducing glomerular hyperfiltration and decreasing tubular transport workload. An epithelial cell-based computational model of a superficial nephron in the rat kidney predicts that reducing glomerular hyperfiltration may decrease tubular transport workload and oxygen consumption, particularly within the proximal convoluted tubules, in diabetic kidneys [110]. Therefore, SGLT-2i may reduce tubular oxygen demand and improve renal oxygenation in the renal cortex by mitigating glomerular hyperfiltration, thereby contributing to the prevention of progressive renal injury [88, 113]. However, the use of SGLT-2i may induce a reduction in medullary kidney oxygenation. In a streptozotocin-induced diabetic rat model, acute SGLT inhibition with phlorizin normalized renal cortical Po2 in diabetic kidneys, whereas medullary Po2 was reduced in both control and diabetic animals [114]. What’s more, a randomized controlled trial involving 120 patients with T2DM and at high risk of CVD followed for 32 weeks demonstrated that EMPA (10 mg/day) significantly increased the value of apparent relaxation rate R2 (corresponding to a reduced oxygenation) in the medulla compared with placebo (25.4 [95% CI 24.7–26.2] Hz in the EMPA group and 24.5 (95% CI 23.9–25.1) Hz in the placebo group), which suggested that EMPA did not improve and even reduced medullary kidney oxygenation compared with placebo in a population with T2DM and normal to slightly reduced kidney function [115].

In addition, emerging evidence suggests that erythrocytosis and the HF benefits produced by SGLT-2i may be related to a shared mechanism [116]. There are different perspectives regarding the physiological mechanism by which gliflozins induce reticulocytosis and erythrocytosis. Sano and Goto proposed that SGLT-2i may allow myofibroblasts to partially revert to erythropoietin-producing fibroblasts by reducing hypoxia in the microenvironment surrounding the proximal tubules, thereby enhancing hematopoiesis and increasing hematocrit [117]. While Heyman et al. have proposed a new perspective that intensified hypoxia at the corticomedullary junction, rather than restored cortical normoxia, mediates gliflozin-associated erythrocytosis [118]. Interestingly, Milton Packer has posited that hepatic sirtuin-1 (SIRT1) upregulation by SGLT-2i stimulates the production of erythropoietin by the liver, potentially representing the shared mechanism coupling erythropoiesis to cardiovascular protection [116].

#### Antioxidant and anti-inflammatory effects

OS is defined as a disruption in the balance between pro-oxidant and antioxidant mechanisms, characterized by excessive production of reactive oxygen species (ROS) and reactive nitrogen species (RNS), which can damage renal cells and contribute to the progression and complications of CKD [6]. In a genetic hypertriglyceridemic rat model, treatment with EMPA for 6 weeks significantly reduced lipoperoxidation products and enhanced the activity of superoxide dismutase, glutathione-dependent enzymes, and glutathione peroxidase, indicating a potential antioxidant role for EMPA in renal protection [119].

Elevated glucose levels promote inflammation by establishing a pro-inflammatory environment [89]. EMPA, as a glucose transport inhibitor, may exert anti-inflammatory effects by reducing hyperglycemia without inducing compensatory increases in SGLT-1 or glucose transporter 2 (GLUT-2) expression, thus mitigating glucose-induced inflammation, particularly in diabetic nephropathy [90]. In a high-glucose-induced oxidative stress model using human renal proximal tubular epithelial cells, hyperglycemia and AGEs suppressed the expression of reversion-inducing cysteine-rich protein with Kazal motifs (RECK) through the oxidative stress/tumor necrosis factor receptor-associated factor 3-interacting protein 2 (TRAF3IP2)/nuclear factor kappa B (NF-κB) and p38 mitogen-activated protein kinase (MAPK)/microRNA-21 (miR-21) pathways. Treatment with EMPA inhibited these pathways, suggesting its potential to alleviate kidney inflammation, fibrosis, and nephropathy under chronic hyperglycemic conditions [120]. In human proximal tubular cells, EMPA significantly suppressed interleukin-1β-induced expression of several inflammatory response genes, including CXCL8/IL8, LOX, NOV, PTX3, and SGK1, further contributing to the renal protective effects of SGLT-2i [121].

### Potential cardioprotective mechanisms

**Fig. 4:**
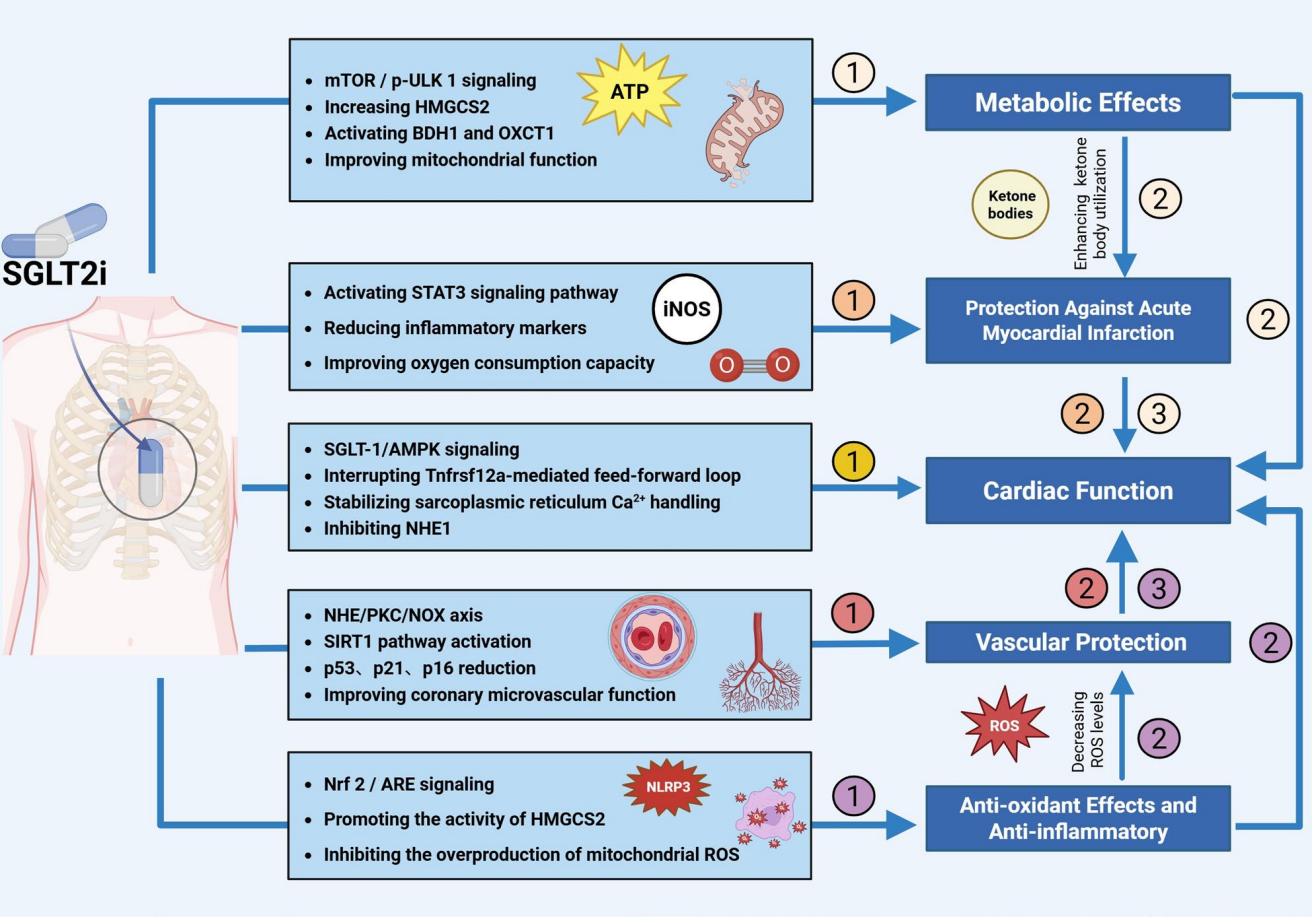
The mechanism of SGLT-2i protecting heart. SGLT-2i exerts cardiovascular protection through multiple interconnected pathways. SGLT-2i protect against the acute myocardial infraction by improving cardiac metabolism via enhancing ketone bodies utilization. These metabolic improvements indirectly enhance cardiac function. Through STAT3 signaling activation, iNOS modulation, and improved oxygen consumption capacity, SGLT-2i protect against the acute myocardial infraction, which further supports cardiac function. SGLT-2i stabilize sarcoplasmic reticulum Ca^2+^ handling, inhibit NHE1, interrupt Tnfrsf12a-mediated feed-forward loops, and modulate SGLT-1/AMPK signaling, thereby enhancing cardiac function. SGLT-2i prevent vascular disease by many ways including SIRT1 pathway activation, reduction of p53, p21, and p16, and improvement of coronary microvascular function. SGLT-2i could be anti-inflammatory by promoting the activity of HMGCS2, inhibiting the overproduction of mitochondrial ROS and activating the Nrf2/ARE signaling. SGLT-2i also protect heart by attenuating NLRP3 inflammasome activation. SGLT-2i, sodium-glucose cotransporter-2 inhibitors; mTOR / p-ULK, monophosphate-activated protein kinase mammalian target of rapamycin/Phosphorylated-Unc-51 Like Kinase 1; HMGCS2: 3-hydroxymethylglutaryl CoA synthetase 2; BDH1: ketolytic enzymes 3-hydroxybutyrate dehydrogenase; OXCT1: 3-oxoacid CoA-transferase1; STAT3: Signal Transducer and Activator of Transcription 3; iNOS: inducible nitric oxide synthase; SGLT-1/AMPK: sodium-glucose cotransporter 1/adenosine monophosphate-activated protein kinase; Tnfrsf12a: tumor necrosis factor receptor superfamily member 12a; NHE1: Na^+^/H^+^ exchanger 1; NHE/PKC/NOX: Sodium Hydrogen Exchanger/Protein Kinase C/Nicotinamide Adenine Dinucleotide Phosphate Oxidase; SIRT1: sirtuin-1; Nrf2/ARE: Nuclear factor erythroid 2-related factor 2/Antioxidant Response Element; NLRP3: NLR family pyrin domain-containing 3; ROS: reactive oxygen species

In contrast to the kidney, SGLT2 is undetectable in normal, ischemic, and hypertrophic human hearts, whereas SGLT1 represents the predominant sodium-glucose cotransporter isoform in cardiomyocytes [122]. Consequently, the cardioprotective effects of SGLT-2i are likely indirect or mediated via alternative targets rather than through cardiomyocyte SGLT2 itself. Acting through these distinct molecular targets, SGLT-2i reduce vascular load, improve diastolic performance, modulate substrate utilization to promote ketone body oxidation, and exhibit anti-inflammatory and antioxidant properties while enhancing vascular function, ultimately conferring overall cardioprotection [123–127] (Fig. 4).

####  Improvement of cardiac function

Vascular load is a critical determinant of ventricular performance [128]. Sustained pathological loading, whether imposed on the right or left ventricle, precipitates maladaptive remodeling and HF [129, 130]. SGLT-2i protect the right ventricle by attenuating pulmonary artery remodeling. In a monocrotaline-induced pulmonary arterial hypertension (PAH) rat model, CANA inhibited the pathological proliferation of pulmonary artery smooth muscle cells via SGLT-1/AMPK signaling modulation, thereby attenuating both pulmonary arterial and right ventricular remodeling [123]. Notably, in this context, CANA exerts cardioprotective effects via direct targeting of SGLT-1 rather than SGLT-2. Similarly, SGLT-2i protect the left ventricle by disrupting pathological feed-forward loops. In a transverse aortic constriction-induced mouse model, EMPA interrupted a tumor necrosis factor receptor superfamily member 12a (Tnfrsf12a)-mediated feed-forward loop that drives left ventricular hypertrophy, thereby attenuating load-induced cardiac stress and ameliorating adverse remodeling [124].

Diastolic dysfunction is increasingly recognized as a major contributor to the development and progression of HF [131]. SGLT-2i may preserve cardiac function by improving diastolic performance. In a db/db mouse model of severe diabetes, EMPA improved left ventricular diastolic function by inhibiting calcium/calmodulin-dependent protein kinase II activation, thereby reducing phosphorylation of the ryanodine receptor, independent of alterations in myocardial ketone or branched-chain amino acid metabolism [132]. This suggests that EMPA improve diastolic function by stabilizing sarcoplasmic reticulum Ca^2+^ handling and preventing diastolic calcium leak. In a non-diabetic porcine HF model, EMPA decreased interstitial myocardial fibrosis, enhanced titin phosphorylation, reduced both left ventricular and cardiomyocyte stiffness, and mitigated histological and molecular remodeling, collectively contributing to improved diastolic function [133]. Although SGLT-2 is not expressed in cardiomyocytes, SGLT-2i can still inhibit Na^+^/H^+^ exchanger 1 (NHE1) in cardiac tissue, potentially via a binding site on NHE1 itself [134]. This inhibition of NHE1 likely represents the key mechanism by which SGLT-2i ameliorate myocardial fibrosis independent of their renal sodium-reabsorptive or blood pressure-lowering effects [134].

#### Protection against acute myocardial infarction

Acute myocardial infarction (AMI) is one of the main causes of cardiovascular death [135]. Among patients hospitalized for AMI, DM is a relevant risk factor and frequent comorbidity, and the duration of DM is parallel to the in-hospital mortality of DM patients hospitalized with AMI [136]. As a novel antidiabetic drug, the chronic treatment with SGLT-2i has a favorable impact on the clinical outcomes of DM patients hospitalized for AMI and may narrow the existing prognostic gap between DM and non-DM patients [137]. The underlying mechanism may be associated with anti-oxidative and anti-inflammatory properties. In a Western diet-fed mouse model of ischemia/reperfusion, EMPA reduced infarct size and improved myocardial function by activating STAT3 signaling pathway, which involved decreasing iNOS expression and subsequent lipid peroxidation [138]. Moreover, EMPA was also shown to prevent kidney function decline in DM patients after AMI [139].

In addition to patients with diabetes, SGLT-2i also confer cardioprotection in non-diabetic individuals with AMI. Li et al. showed that DAPA significantly improve cardiac function and reduce the occurrence of adverse cardiovascular events in patients with myocardial infarction, independent of diabetes status [140]. In addition, in non-diabetic patients experiencing acute ST-segment elevation myocardial infarction and undergoing percutaneous coronary intervention, EMPA may effectively reduce inflammatory markers and improve oxygen consumption capacity, thereby having a cardioprotective effect [141]. The cardioprotective effects of SGLT-2i in non-diabetic AMI patients may be mediated through cardiac metabolic mechanisms (e.g., enhanced ketone body utilization). In a non-diabetic porcine model of ischemia/reperfusion, EMPA significantly improved myocardial salvage and preserved cardiac function by increasing circulating ketone body levels and facilitating their preferential myocardial utilization as an energy substrate [142].

#### Cardiac metabolic effects

Under normal physiological conditions, cardiac energy production predominantly relies on the oxidation of fatty acids. However, in patients with cardiomyopathy and HF, the myocardium exhibits a shift toward glucose utilization rather than fatty acid oxidation [143]. SGLT-2i may exert cardioprotective effects by shifting substrate preference back toward ketone bodies and regulating the metabolism of glucose and branched-chain amino acids (BCAA), while simultaneously improving mitochondrial function.

Ketone bodies serve as a metabolic fuel for the energy-starved heart [144]. SGLT-2i facilitate this protective metabolic shift by modulating the enzymes related to ketone homeostasis. An integrated analysis of nine Gene Expression Omnibus (GEO) datasets related to diabetic cardiomyopathy (DCM) revealed increased activity of the 3-hydroxy-3-methylglutaryl-CoA synthase 2 (HMGCS2) gene in DCM hearts, alongside suppressed expression of the ketolytic enzymes 3-hydroxybutyrate dehydrogenase (BDH1) and 3-oxoacid CoA-transferase 1 (OXCT1) [125]. EMPA restored this balance by enhancing BDH1/OXCT1-mediated myocardial ketone oxidation and promoting HMGCS2-driven ketogenesis, thereby increasing the utilization of ketone bodies [125]. What’s more, EMPA prevented mitochondrial breakage and dysfunction, and increased myocardial ATP to provide sufficient energy, ultimately ameliorating cardiac dysfunction in DCM [125]. These findings suggest that EMPA may serve as a therapeutic option for DCM by optimizing cardiac energy metabolism.

Beyond ketone body oxidation, SGLT-2i further protect the heart through integrated regulation of glycolysis and BCAA metabolism. In a transverse aortic constriction-induced HF mouse model, EMPA bound to cardiac glucose transporters to inhibit glycolysis, reestablish the coupling between glycolysis and oxidative phosphorylation, and regulate the adenosine monophosphate-activated protein kinase/mammalian target of rapamycin complex 1 (AMPK/mTORC1) signaling pathway, thereby attenuating left ventricular remodeling and pressure-overload HF [145]. Additionally, increasing evidences indicate that SGLT-2i may also regulate BCAA metabolism, thus contributing to their cardioprotective effects. In a DCM mouse model, EMPA promoted BCAA catabolism and inhibited the mTOR/phosphorylated-Unc-51 like kinase 1 signaling pathway, thereby restoring autophagic activity—typically suppressed in diabetes—and alleviating diabetes-related myocardial injury [146].

#### Antioxidant and anti-inflammatory effects

OS, defined as an imbalance between ROS production and the endogenous antioxidant defense system, plays a critical role in the development and progression of HF [147]. SGLT-2i counteract cardiac OS through multiple pathways. In a diabetic rat model, EMPA reduced mitochondrial ROS generation in the atrium via activation of AMPK signaling pathways, thereby alleviating myocardial OS and fibrosis and improving cardiac function [126]. Similarly, in a diabetic mouse model, EMPA ameliorated myocardial OS and fibrosis by inhibiting the transforming growth factor β (TGF-β)/Smad signaling pathway and activating the nuclear factor erythroid 2-related factor 2/antioxidant response element (Nrf2/ARE) signaling pathway, ultimately enhancing cardiac structure and function [148].

Impaired regulation of inflammatory and anti-inflammatory mechanisms may result in defective tissue repair, sustained injury, and eventual HF [149]. Beyond antioxidant effects, SGLT-2i exert cardioprotective effects by modulating the inflammatory milieu. Given the established enhancement of HMGCS2 activity by SGLT-2i in diabetic cardiomyopathy [125], these agents may confer additional myocardial protection by promoting M2 macrophage polarization via the phosphatidylinositol 3-kinase/protein kinase B (PI3K/Akt) signaling pathway, thereby suppressing pro-inflammatory macrophage activation and supporting an anti-inflammatory phenotype, ultimately attenuating sepsis-induced myocardial injury [150].

As a regulatory node of oxidative stress and inflammatory diseases, the nucleotide-binding domain-like receptor protein 3 (NLRP3) inflammasome plays a critical role in the pathogenesis of cardiovascular diseases [151]. In rodent models of HF, EMPA reduced NLRP3 inflammasome activation and decreased the expression of sterile inflammation-related markers in cardiac tissue [127]. These effects led to reductions in cardiac inflammation and preservation of cardiac function, and were mediated through a calcium-dependent mechanism, independent of alterations in circulating ketone bodies, myocardial ketone oxidation, or cardiac ATP production [127]. These findings suggest that EMPA may offer therapeutic benefits in HF irrespective of the presence of diabetes [127].

#### Vascular protection effects

Endothelial dysfunction is closely associated with major cardiovascular risk factors [152]. SGLT-2i may contribute to protecting the heart by improving endothelial function. In human coronary arterial endothelial cells under mechanical stress (the exposure to cyclic stretch), EMPA attenuated the activation of the Sodium Hydrogen Exchanger/Protein Kinase C/Nicotinamide Adenine Dinucleotide Phosphate Oxidase (NHE/PKC/NOX) axis, thereby reducing ROS production and ameliorating endothelial damage [153]. Additionally, SGLT-2i ameliorate endothelial dysfunction by downregulating the expression of endothelial senescence markers (p53, p21, p16) in obese ZSF1 rats [154] and increasing nitric oxide bioavailability and decreasing ROS levels via activation of the SIRT1 pathway in senescent endothelial cells from T2DM mice [155]. The coronary arteries supply oxygen to the myocardium to meet the oxygen demands of myocardial cells for their normal physiological activities [156]. In the streptozotocin-induced diabetic rat model, EMPA induced coronary artery relaxation by activating Large conductance calcium-activated K channel through a Sirtuin 1-Nuclear factor erythroid 2-related factor 2 (Sirt1-Nrf2) mechanism, thereby inducing coronary vasodilation in diabetes [157], suggesting that EMPA may improve cardiac function by inducing coronary artery relaxation. Furthermore, in the prediabetic ob/ob^(−/−)^ mouse model, EMPA improved coronary microvascular function and cardiac contractility by inducing a catabolic state characterized by reduced blood cholesterol and Hemoglobin A1c (HbA1c), increased glucagon/insulin ratio and ketone levels, and improved endothelial function markers such as L-arginine/ADMA ratio, thereby enhancing coronary flow velocity reserve and fractional area change [158].

## Therapeutic evidence and clinical application of SGLT-2i in CKM

SGLT-2i function by inhibiting the reabsorption of glucose and sodium in the renal proximal tubules, thereby promoting urinary glucose excretion [159]. Initially, developed as hypoglycemic agents for T2DM management, they have since been found to exert significant renoprotective and cardioprotective effects [11, 12]. In a recent large-scale meta-analysis (with 70,361 participants and 10 placebo-controlled trials), SGLT-2i significantly reduced the risk of CKD progression and renal failure, and demonstrated consistent benefits across different baseline renal functions (eGFR) and albuminuria conditions [160]. These clinical effects are consistent with the underlying mechanism: by restoring the regulatory feedback between the glomerulus and the distal part (i.e., improving the hemodynamic parameters of the proximal tubule), reducing intraglomerular pressure, reversing the adverse hyperfiltration state, and thereby minimizing renal unit damage. SGLT-2i uniquely target the interrelated pathophysiology of metabolic, renal, and cardiovascular diseases, establishing their role as cornerstone therapy for CKM syndrome. A comprehensive systematic review and meta-analysis of 15 RCTs with 100,952 patients across the cardiometabolic disease spectrum provides compelling evidence supporting the transition of SGLT-2i from glucocentric agents to foundational cardiorenal protective drugs. This comprehensive analysis demonstrated that SGLT-2i significantly reduced the risk of hospitalization for heart failure (HHF) by 29% (HR 0.71, 95% CI 0.67–0.77) and cardiovascular death by 14% (HR 0.86, 95% CI 0.79–0.93) across a broad spectrum of patients with cardiometabolic disease, including those with T2DM, established atherosclerotic cardiovascular disease, HF, and CKD. The robustness of these findings is underscored by the remarkably low statistical heterogeneity (e.g., I² = 0% for HHF), indicating a highly consistent treatment effect across all patient subgroups and individual trials. The narrow confidence intervals for the primary outcomes reflect a high degree of precision in the effect estimates. While limitations such as the study-level (vs. individual-patient-level) meta-analysis design exist, the results were derived from high-quality RCTs, with funnel plots showing no significant publication bias. The consistent and relatively precise cardiorenal benefits observed across a range of glycemic statuses support a class effect that appears to extend beyond glucose lowering, thereby suggesting SGLT-2i as an important component of contemporary cardiorenal therapy [161]. The ADA and the European Association for the Study of Diabetes recommend SGLT-2i for patients with T2DM who have established CVD or CKD [162]. The 2022 Kidney Disease: Improving Global Outcomes (KDIGO) Clinical Practice Guideline for Diabetes Management in CKD recommends lowering the eGFR threshold for initiating SGLT-2i and endorses SGLT-2i as first-line therapy in patients with T2DM and CKD [163]. DAPA and EMPA have recently been approved by the Food and Drug Administration for the treatment of HF [164].

### Stage 0: CKM risk factors

The population at CKM stage 0 exhibits no identifiable risk factors. For this group, the emphasis lies in primordial prevention by maintaining normal anthropometric indices, blood glucose, blood pressure, and lipid profiles to minimize the risk of CKD or CVD [9]. A common early risk factor for CKM syndrome is obesity, which frequently coexists with metabolic dysregulation and contributes to the development of cardiovascular and renal complications. Notably, the earliest determinant of obesity is the intrauterine environment, which is strongly influenced by maternal health during pregnancy [7]. Therefore, close monitoring and guidance for women during pregnancy and childbirth are essential [165].

During childhood, it is recommended that body mass index (BMI) be assessed at least annually, with age-appropriate dietary and physical activity guidance provided to help prevent pediatric obesity [9, 166]. For adults at CKM stage 0, particularly those with multiple metabolic or cardiovascular risk factors, early intervention strategies such as lifestyle modification and dietary regulation are advised to prevent the emergence of CKM-associated risks. Regular health examinations, increased awareness of CKM risk factors, understanding of CKM staging, and targeted preventive measures are necessary. Particular attention should be given to obesity by monitoring both BMI and waist circumference.

### Stage 1: excessive or dysfunctional obesity

The primary therapeutic objective at CKM stage 1 is to address abnormal adiposity, normalize body weight, prevent the onset of metabolic disorders, and reduce associated risk factors.

Weight reduction through physical activity and lifestyle modification remains the first-line strategy. Pharmacologic interventions are considered when lifestyle modifications are insufficient. Many commercial weight-loss agents have high adverse event rates and frequently fail to meet regulatory standards [167]. Several antidiabetic medications—such as biguanides (e.g., metformin), glucagon-like peptide-1 receptor agonists (GLP-1 RAs), and SGLT-2i—have been recognized for their weight-reducing effects [24]. According to the EASO guidelines, when choosing a medication, one should consider its effect on weight loss, its impact on complications, and its safety. The evidence from the real world supports the effectiveness of GLP-1RA in weight loss. However, in general, adverse events in obese individuals, especially long-term safety outcomes, have not been fully studied [168]. For patients with severe obesity, bariatric (metabolic) surgery may be considered and has demonstrated clinical efficacy [169].

SGLT-2i offers a therapeutic advantage in weight management by regulating energy balance and inducing metabolic changes [170]. A clinical study conducted by Szekeres et al. (*N* = 102) demonstrated that diabetic and obese patients treated with EMPA experienced significant reductions in body weight, fat mass, leptin levels, urea nitrogen, and creatinine. Beyond their established cardio-renal protective properties, SGLT-2i may also modulate leptin resistance [171]. A retrospective cohort study of 1,520 patients with T2DM in Canada revealed that individuals lost approximately 2.2 ± 3.1 kg of body weight after 3 to 6 months of DAPA monotherapy, relative to baseline (*P* < 0.01) [172]. Additionally, a meta-analysis included 55 RCTs, that examined the linear trends of dose and weight changes of six SGLT-2is. The results showed that all six different doses of SGLT-2i could lead to significant weight loss, and the weight loss in patients treated with DAPA was dose dependent. When DAPA was compared with a placebo, DAPA 2.5 mg, DAPA 5 mg, 10 mg, and 20 mg led to significantly greater changes in weight (WMD, − 1.30 kg, 1–1.51 kg, − 1.79 kg, and − 2.24 kg, respectively; *P* < 0.00) [173]. Collectively, these findings suggest that SGLT-2i may be effectively utilized in CKM stage 1 patients to promote weight loss, reduce adiposity, prevent obesity-related metabolic disorders, and delay disease progression.

### Stage 2: metabolic risk factors or CKD

The primary therapeutic goals at CKM stage 2 include correcting metabolic abnormalities, treating diabetes and CKD aggressively, and preventing progression to end-stage renal disease and CVD [174].

SGLT-2i influence systemic metabolism by altering energy homeostasis [170]. They lower blood glucose by inhibiting renal glucose reabsorption in the proximal tubules, promoting urinary glucose excretion. After SGLT-2i administration, the glucose excreted in the urine translates to approximately 200–300 calories each day [175]. This mechanism contributes to weight loss and carries a lower risk of hypoglycemia compared to other antidiabetic agents [159]. As such, SGLT-2i have emerged as a preferred treatment option and are often used in combination with other glucose-lowering therapies. Notably, SGLT-2i are under investigation for the treatment of NAFLD and have demonstrated efficacy in vitro, in animal models, and in clinical studies [55, 176].

Renal impairment is also a frequent consequence of progressive metabolic disease, particularly in the transition to CVD and HF [177, 178]. Substantial evidence supports the nephroprotective properties of SGLT-2i [179, 180], with efficacy demonstrated across various stages of CKD [181].

In the EMPA-KIDNEY trial, among 6,609 participants who were assigned to empagliflozin (EMPA) or placebo 10 mg once daily, the EMPA group exhibited lower progression of nephropathy or death from cardiovascular causes after 2.0 years of follow-up (HR 0.72, 95% CI 0.64–0.82; *P* < 0.001), as well as a lower hospitalization rate compared to the placebo group (HR 0.86, 95% CI 0.78–0.95; *P* = 0.003) [182], and no significant difference was observed in the baseline cardiovascular risk spectrum [183]. Further analysis of the trial demonstrated that EMPA reduced the risk of nephropathy progression (risk ratio 0.71, 95% CI 0.62–0.81), leading to a slight acute decline in eGFR, followed by a significant slowing of long-term progression in chronic kidney disease [184]. The relative effects were similar in analyses of different primary kidney diseases [185]. Additional cardiorenal benefits were observed for up to 12 months after discontinuation (risk ratio 0.79, 95% CI 0.72–0.87) [7], indicating that SGLT-2i should be included as part of standard therapy to reduce the risk of renal failure in chronic kidney disease. Similarly, in the DAPA-CKD trial, dapagliflozin (DAPA) also reduced the risk of major renal and cardiovascular adverse events and all-cause mortality in patients with diabetes and non-diabetic CKD [102, 186].

In the CREDENCE trial, the relative risk of the primary outcome was 30% lower in the CANA group than in the placebo group, with a 34% reduction in the renal-specific composite of end-stage renal disease, doubling of creatinine levels, or renal death (risk ratio 0.66, 95% CI 0.53–0.81; *P* < 0.001). The CANA group also demonstrated lower risks of cardiovascular death, myocardial infarction (MI), and stroke (risk ratio 0.80, 95% CI 0.67–0.95; *P* = 0.01) and lower hospitalization rates for HF (HR 0.61, 95% CI 0.47–0.80; *P* < 0.001). These results suggest that CANA may be an effective therapeutic option for renal and cardiovascular protection in patients with T2DM and CKD [14].

A meta-analysis incorporating 13 RCTs with 90,413 participants showed that SGLT-2i reduced the risk of CKD progression by 37% (relative risk [RR] 0.63, 95% CI 0.58–0.69) and the risk of acute kidney injury by 23% (RR 0.77, 95% CI 0.70–0.84). Moreover, they reduced the risk of cardiovascular death or hospitalization for HF by 23% (RR 0.77, 95% CI 0.74–0.81), and cardiovascular mortality alone by 14% (RR 0.86, 95% CI 0.81–0.92) [187]. Additionally, SGLT-2i possess antihypertensive properties, further contributing to their cardiovascular benefits [159].

A cohort study involving 36,476 patients with T2DM and CKD found that those treated with SGLT-2i had a significantly lower risk of non-fatal myocardial infarction or stroke (HR 0.86, 95% CI 0.78–0.94) [188]. Additionally, a systematic review and network meta-analysis comprising eight studies and 36,186 patients with T2DM and CKD revealed that combination therapy with SGLT-2i and mineralocorticoid receptor antagonists (MRAs) significantly improved cardiovascular outcomes compared with monotherapy or placebo (RR [95% CI]: vs. SGLT-2i, 0.76 [0.60–0.96]; vs. MRAs, 0.66 [0.53–0.82]; vs. placebo, 0.58 [0.47–0.73]) [189].

Owing to their numerous clinical benefits, the ADA and the European Association for the Study of Diabetes have recommended SGLT-2i as first-line therapy in patients with T2DM and comorbid CVD or kidney disease [190]. In light of their renoprotective and cardioprotective effects, patients with CKD should be considered for priority treatment with SGLT-2i [9]. The evidence discussed above supports the application of SGLT-2i in managing CKM stage 2, with potential benefits including the attenuation of disease progression, reduction of morbidity, and improvement of long-term prognosis.

### Stage 3: subclinical cardiovascular disease in CKM syndrome

Patients at CKM stage 3 require prophylactic treatment to identify those at high risk for CVD and to prevent progression to overt CVD and renal failure. Hypertension is the most prevalent complication of CKD and is associated with an increased risk of end-stage renal disease, cardiovascular events, and mortality. SGLT-2i, in addition to their diuretic properties, modulate fluid homeostasis and regulate blood pressure in CKD patients, playing a critical role in the prevention of both CVD and end-stage renal failure [191]. A randomized controlled trial involving CKD patients (*n* = 24) demonstrated that SGLT-2i, in combination with angiotensin-converting enzyme inhibitors (ACEIs), significantly modulated the renin-angiotensin-aldosterone system (RAAS), thereby enhancing angiotensin regulation in diabetic nephropathy [192]. In the CVD-REAL trial, the mortality rate of HF was compared between patients treated with SGLT-2i and those treated with other hypoglycemic agents. The incidence of HF was lower (risk ratio 0.61, 95% CI 0.51–0.73), and there was a reduction in both HF and mortality (HR 0.54, 95% CI 0.48–0.60) [193].

The DECLARE-TIMI 58 trial, which evaluated 17,160 patients with T2DM who were randomly assigned to receive either DAPA or placebo, reported a median follow-up period of 4.2 years. The results demonstrated a lower rate of HHF in the DAPA group (HR 0.73, 95% CI 0.61–0.88), and a reduced incidence of renal events (4.3% in the DAPA group vs. 5.6% in the placebo group; HR 0.76, 95% CI 0.67–0.87) [15].

A meta-analysis involving 31 RCTs and four cohort studies (*n* = 4,322) of patients with T2DM and/or CVD indicated that among newer hypoglycemic agents, SGLT-2is were most effective in improving ventricular remodeling outcomes [194]. Although many individuals with T2DM receive prophylactic treatment for CVD, they remain at substantial risk of developing ASCVD. Recently, attention has focused on the cardiovascular protective properties of SGLT-2i [195]. A meta-analysis of 18 RCTs found that SGLT-2i significantly reduced arterial stiffness, as evidenced by a decrease in pulse wave velocity (mean difference = -0.76, 95% CI -1.45 – -0.08) [196]. Inhibition of early atherosclerotic progression is of great significance in CVD prevention [197]. The administration of SGLT-2i in T2DM patients reduces the incidence of HHF and cardiovascular events [198]. For individuals at risk of HF within CKM syndrome, prioritizing SGLT-2i therapy is advisable [9].

Furthermore, a meta-analysis encompassing eight large RCTs with 16,460 HF patients demonstrated that SGLT-2i conferred significant cardiovascular benefits in the majority of patients, irrespective of diabetes status [199]. SGLT-2i are increasingly considered the preferred therapeutic agents for T2DM patients with existing or potential HF [198]. Current guidelines strongly recommend SGLT-2i for patients with established CVD and CKD [200].

### Stage 4: CKM syndrome and clinical cardiovascular disease

Many patients at CKM stage 4 also suffer from renal failure and may require long-term renal replacement therapy [9]. This underscores the value of SGLT-2i as cardio-renal protective agents. Multidisciplinary approaches involving combination pharmacotherapy, regular monitoring, prognostic assessment, and integrated care can improve survival outcomes and prolong life expectancy in CKM patients.

Numerous cardiovascular and renal outcome trials have consistently shown that SGLT-2i are associated with significant reductions in major adverse cardiovascular events (MACE), HHF, all-cause mortality, and the progression of renal dysfunction [99, 100, 102] (Table 3). In patients with HF with preserved ejection fraction (HFpEF), SGLT-2i significantly reduced the composite risk of cardiovascular death or HHF [201, 202].

In the EMPULSE trial, 530 patients with chronic HF were randomly assigned to receive once-daily EMPA 10 mg or placebo, with a maximum treatment duration of 90 days. The results demonstrated that compared to placebo, patients treated with EMPA exhibited better tolerability and a higher proportion of those who achieved clinical benefits [203]. The PRESERVED-HF trial demonstrated that 12 weeks of DAPA treatment significantly improved patient-reported symptoms, physical limitations, and motor function [204].

In the EMPEROR-Reduced trial, 3,730 patients with HF received placebo or EMPA in a double-blind design, with a median treatment duration of 16 months. EMPA reduced the risk of death, hospitalization for heart failure, or emergency heart failure visits requiring intravenous therapy (415 versus 519 patients; EMPA versus placebo, respectively; HR 0.76, 95% CI 0.67–0.87; *P* < 0.0001) [205]. Clinical evidence also supports the antihypertensive effects of SGLT-2i. In a randomized clinical trial involving patients with HFpEF (*N* = 5533, follow-up of up to 172 weeks), the EMPA group (10 mg once daily) experienced a greater reduction in systolic blood pressure than the placebo group. Especially in patients with resistant hypertension at weeks 4–32, there was a significant treatment difference (*P* = 0.001–0.009) with a mean difference in SBP between 2.4 and 3.3 mmHg [206].

In the DAPA-HF trial, patients with HF and reduced ejection fraction who received DAPA treatment had a lower risk of cardiovascular-related heart failure or death compared to those receiving placebo, regardless of whether they had T2DM [99].

In the DEFORM trial, 104 patients with functional mitral regurgitation were randomly assigned in a 1:1 ratio to receive DAPA (DAPA; 10 mg/day) or no treatment for 3 months. DAPA significantly reduced mitral regurgitation and improved myocardial remodeling, further supporting the role of SGLT-2i in managing HF [207].

The EMPA-REG OUTCOME trial showed that EMPA significantly reduced cardiovascular mortality, HHF, and progression of kidney disease in patients with T2DM and established CVD [16]. Cardiovascular and renal outcome trials have demonstrated that SGLT-2i are associated with significant reductions in MACE, HHF, all-cause mortality, and the progression of worsening renal function [99, 100, 102]. Additionally, in patients with HFpEF, SGLT-2i significantly reduce the composite risk of cardiovascular death or HHF [201, 202].

A comprehensive systematic review and meta-analysis evaluating the impact of SGLT-2i on HF outcomes and cardiovascular mortality analyzed data from 15 RCTs (*N* = 100,952) across the cardiometabolic disease spectrum. The analysis demonstrated consistent benefits of SGLT-2i therapy in reducing HF events and cardiovascular deaths in patients with HF, T2DM, CKD and atherosclerotic cardiovascular disease [161]. A narrative review further affirmed the utility of SGLT-2i in both the treatment and prevention of HF [208]. The 2021 European Society of Cardiology Guidelines recommend SGLT-2i to reduce hospitalization for HF, MACE, end-stage renal disease, and cardiovascular mortality in patients with T2DM. The guidelines also emphasize the importance of individualized therapeutic strategies for patients with or at risk of CVD [209–211].

**Table 3:** Clinical trials of SGLT-2i for the treatment of CKM syndrome

Trial	Disease	NCT number	Trial description	Interventions	Age	Phases of CKM	Enrollment	Primary efficacy outcome	Ref
CREDENCE	T2DM and CKD	NCT02065791	CANA and renal outcomes in T2DM and nephropath	CANA placebo 100 mg once daily	Average 63	2	4401	The primary composite outcomes, including the risk of serum creatinine doubling or death from renal or cardiovascular causes, the risk of end-stage renal disease, the risk of hospitalization for HF, and the composite risk of cardiovascular death, MI, or stroke, were all reduced.	[14]
DECLARE-TIMI 58	T2DM and ASCVD	NCT01730534	DAPA and cardiovascular outcomes in T2DM	DAPA or placebo 10 mg once daily	57–71	3	17,160	Reduced the rate of cardiovascular death or hospitalization due to HF	[15]
EMPA-REG	T2DM and high cardiovascular risk	NCT01131676	Effect of EMPA on cardiovascular outcomes and mortality in patients with T2DM and high cardiovascular risk	10 mg of EMPA, 25 mg of EMPA , or placebo once daily	54–72	4	7020	The primary composite outcome of mortality from cardiovascular causes, non-fatal MI, or non-fatal stroke was lower in patients receiving EMPA compared to those receiving the placebo.	[16]
DAPA-HF	patients with HF and reduced ejection fraction	NCT03036124.	DAPA in patients with HF and reduced ejection fraction	DAPA or placebo 10 mg once daily	> 18	4	4744	Reduced incidence of primary outcome and increased the risk of HF	[99]
DAPA-CKD	CKD	NCT03036150	Effects of DAPA on major adverse kidney and cardiovascular events in patients with diabetic and non-diabetic CKD	DAPA or or placebo 10 mg once daily	20–75	2	4304	Reduced the risk of major renal and cardiovascular adverse events and all-cause mortality in patients with diabetes and non-diabetic CKD	[102]
EMPA-KIDNEY	CKD	NCT03594110	EMPA in patients with CKD	EMPA or placebo 10 mg once daily	50–78	2	6609	Reduced the risk of kidney disease progression or death due to cardiovascular	[182]
EMPA-REG OUTCOME	T2DM and ASCVD	NCT01131676	EMPA reduced mortality and HHF across the spectrum of cardiovascular risk	10 mg of EMPA, 25 mg of EMPA, or placebo once daily	54–72	2	7020	Across subgroups, the relative reductions in cardiovascular mortality risk, all-cause mortality, 3-point MACE, and hospitalization risk for HF were consistent.	[183]
EMPA-KIDNEY	CKD	NCT03594110	Effects of EMPA on renal outcomes and eGFR slope in participants with different types of kidney disease	EMPA or placebo 10 mg once daily	Average 65	2	6609	The early stage of eGFR shows a slight acute decline, followed by a significant slowing of the long-term progression of CKD.	[184]
EMPA-KIDNEY	CKD	NCT03594110	Impact of primary kidney disease on the effects of EMPA in patients with CKD	EMPA or placebo 10 mg once daily	Average 65	2	6609	The relative effects were similar in analyses of different primary kidney diseases	[185]
DAPA-CKD	CKD	NCT03036150	DAPA in patients with CKD	DAPA or or placebo 10 mg once daily	20–75	2	4304	A persistent decline in GFR of at least 50%, end-stage renal disease, or a reduced risk of death due to renal or cardiovascular causes	[186]
CVD-REAL	Patients using SGLT-2i or other hypoglycemic agents	NCT02993614	Comparative effectiveness of cardiovascular outcomes in users of SGLT-2i	–	Average 57	3	1,392,254	The composite end-point mortality rate for HHF or all-cause death was reduced by 51% or 46%.	[193]
EMPULSE	HF	NCT04157751	The SGLT-2i EMPA in patients hospitalized for acute HF	EMPA or placebo 10 mg once daily	61–78	4	530	Improvement of survival, reduction of HFEs, and improvement of symptoms	[203]
PRESERVED-HF	HF with HFpEF	NCT03030235	The SGLT-2i DAPA improve the symptoms, physical limitations and motor function of patients with phenotypic HFpEF	DAPA or placebo 10 mg once daily	63–77	4	324	Improved KCCQ-CS, improvement in 6MWT distance, resulted in greater weight loss	[204]
EMPEROR-Reduced	HF	NCT03057977	Effect of EMPA on the clinical stability of patients with HF and a reduced ejection fraction	EMPA or placebo 10 mg once daily	65	4	3730	Reduced the risk of cardiovascular death or HHF	[205]
EMPEROR-Preserved	Hypertension and HF	NCT03057951	The effects of EMPA on systolic blood pressure (SBP), time in target range, incidence of hypertensive urgencies	EMPA or placebo 10 mg once daily	62–82	4	5533	Reduced incident hypertensive urgencies; reduced incident hypertensive urgencies	[206]
DEFORM	FMR	NCT05606718	DAPA effect on functional mitral regurgitation and myocardial remodeling	DAPA or placebo 10 mg once daily	49–77	4	104	Reduced the degree of mitral regurgitation, improved cardiac function, and promoted myocardial remodeling	[207]

T2DM: Type 2 Diabetes; CKD: Chronic Kidney Disease; CANA: canagliflozin; HF: heart failure; MI: myocardial infarction; ASCVD: atherosclerotic CVD; DAPA: dapagliflozin; EMPA: empagliflozin; HHF: hospitalization for heart failure; MACE: major adverse cardiovascular events; eGFR: estimated glomerular filtration rate; SGLT-2i: sodium-glucose cotransporter-2 inhibitors; FMR: Functional Mitral Regurgitation; HFpEF: heart failure with preserved ejection fraction

## Possible adverse effects and the safety of SGLT-2i

Although SGLT-2i demonstrate significant benefits in metabolic, renal, and cardiovascular protection, certain common adverse effects warrant attention. Clinical practice guidelines for SGLT-2i used in adult patients with T2DM have identified several associated adverse effects, including diabetic ketoacidosis, and genital infection [200]. A large number of systematic reviews and meta-analyses have evaluated the adverse effects related to the use of SGLT-2is. A comprehensive systematic review and network meta-analysis analyzed the benefits and harms of drug treatment for type 2 diabetes, including the following data of SGLT-2i: genital infections: analysis of 94 trials (*N* = 103,111) demonstrated that SGLT-2i significantly increased the risk of genital infections (OR 3.30, 95% CI 2.88–3.78); amputation risk: evaluation of 18 trials (*N* = 107,503) suggested a potential increased risk of amputation (OR 1.27, 95% CI 1.01–1.61); DKA: assessment of 36 trials (*N* = 138,322) revealed a heightened risk of diabetic ketoacidosis (OR 2.07, 95% CI 1.44–2.98) [212]. In persons with T2DM and ASCVD, the adverse effects of SGLT-2i also contribute to treatment discontinuation as a secondary reason [213].

### Genital mycotic infections

SGLT-2i inhibit glucose reabsorption in the renal tubules, leading to elevated urinary glucose levels. This may create a favorable environment for the growth of other commensal genital microorganisms, increasing the risk of bacterial and fungal infections in the genitourinary tract [214]. Studies indicate that patients with T2DM treated with SGLT-2i have a risk of genital fungal infections (such as vulvovaginitis and balanitis), particularly in women [215–217]. Various clinical trial data indicate that the use of SGLT-2i in the treatment of T2DM is associated with an increased risk of genital infection and, to a lesser extent, an increased risk of urinary tract infection [214]. A meta-analysis involving 77 RCTs with a total of 50,820 participants indicated that SGLT-2i increased the risk of genital infections (RR 3.30, 95% CI 2.74–3.99; moderate quality evidence) [218]. Although these infections are generally mild, they may occasionally lead to more severe complications such as cystitis, pyelonephritis, and urosepsis.

### Acute kidney injury (AKI)

Contrast agent-induced acute kidney injury (CI-AKI) is an important complication for patients with T2DM and renal insufficiency who undergo percutaneous coronary intervention (PCI). A retrospective study aimed to evaluate the renal protective effect of SGLT-2i to prevent CI-AKI in patients with HF undergoing invasive surgery with iodinated contrast media (ICM). A total of 86 patients receiving SGLT-2i treatment and 179 patients not receiving SGLT-2i treatment were included. The results showed that the incidence of CI-AKI in the SGLT-2i group was lower than that in the non-users group (9.3% vs. 27.3%, *p* < 0.001), and SGLT-2i treatment was associated with a reduced risk of CI-AKI in patients with HF undergoing ICM invasive surgery (OR: 0.41, 95% CI: 0.16–0.90, *p* = 0.045) [219].

Although some evidence suggested that SGLT-2i has a positive effect on AKI, in high-risk patients undergoing PCI, the risk of contrast-induced AKI may increase if the contrast agent is administered shortly before exposure. A recent study included 354 patients with T2DM and renal insufficiency who underwent PCI. Among them, 183 patients used DAPA shortly before PCI, while 171 patients were in the control group. The median duration of short-term use of DAPA was 3 days (2, 6), with an average of 3.56 ± 1.62 days. The study found that short-term use of DAPA before PCI was associated with an increased risk of CI-AKI (OR = 2.308, 95% CI: 1.002–5.314, *p* = 0.049), indicating that the timing of its use should be carefully evaluated in high-risk patients [220]. Therefore, at different time points and in different clinical contexts, the concept of “AKI risk” should be carefully defined. Long-term use of SGLT-2i may reverse the high filtration state, reduce oxygen consumption, improve factors outside the kidneys, such as cardiac function, sympathetic nerve activity, and systemic hemodynamics; and stimulate the “hypoxic adaptation” mechanism in the renal medulla mediated by hypoxia-inducible factor (HIF), thereby helping to maintain the energy homeostasis of renal tissue and resist oxidative damage, while supporting cell repair [221]. However, the importance of timing cannot be ignored. Starting to use SGLT-2i in the short term (such as a few days before surgery) may increase the risk of AKI, especially in patients with unstable hemodynamics, insufficient volume, or poor renal function. This may be because the protective regulatory mechanisms formed during chronic use have not been activated, and the early osmotic diuresis effect and changes in blood volume may lead to mild hypovolemia, thereby inducing the manifestations of prerenal AKI [222].

A systematic review and meta- analysis included 11 RCTs related to SGLT-2i, involving a total of 58,534 participants. This study found that in the initial 2–4 weeks, compared with the placebo group, eGFR in the SGLT-2i group decreased acutely (weighted mean difference [WMD] -3.35 mL/min/1.73 m2 ; 95% CI, -3.81 to -2.90). After that, the renal protective effect gradually emerged [223]. The post-hoc analysis of the EMPA-REG OUTCOME trial demonstrated that the initial “eGFR decline” had little impact on the therapeutic effects of EMPA on subsequent cardiovascular death, HHF, and renal disease exacerbation [224, 225]. It is worth noting that the early “eGFR decline” caused by SGLT-2i is a hemodynamic adjustment response rather than tissue damage, and therefore should not be simply regarded as a marker of renal injury.

Although SGLT-2i exhibit renal protective effects and slow the decline of renal function in T2DM patients, dose adjustment or discontinuation may be necessary if renal function worsens. SGLT-2i are contraindicated in patients with impaired renal function and an eGFR below 30 mL/min/1.73 m². For CKD patients with an eGFR below 60 mL/min/1.73 m², renal function should be closely monitored during SGLT-2i therapy [226].

### DKA

A rare but serious adverse effect of SGLT-2i is the development of euglycemic DKA. This condition is characterized by normal or mildly elevated blood glucose levels, complicating diagnosis and necessitating heightened vigilance among healthcare providers [227]. The underlying mechanisms, as summarized in the literature, may involve increased ketone body production due to a shift in substrate utilization toward fatty acids, enhanced ketone reabsorption, delayed ketone clearance, and weight loss accompanied by reduced muscle mass. Additionally, adverse metabolic conditions, dehydration, gastric fluid loss, and infections may trigger the progression of this condition, predisposing patients to ketosis [228, 229]. Therefore, when prescribing SGLT-2i, clinicians should educate patients on the signs and symptoms of DKA, such as nausea, vomiting, abdominal pain, and weakness, even in the presence of normal blood glucose levels.

### Fractures and amputations

Some studies suggest that CANA may increase the risk of fractures, potentially due to alterations in calcium and phosphate homeostasis or an increased risk of falls, necessitating further research to establish a definitive link [230]. The CANVAS trial reported an elevated risk of lower limb amputations with CANA, a specific SGLT-2i (HR 1.97, 95% CI 1.41–2.75). This finding has not been consistently observed with other agents in the class, indicating a possible drug-specific effect [13, 231].

### The safety of SGLT-2i

While SGLT-2i demonstrate broad cardiorenal and metabolic benefits, their safety profile in specific populations, such as elderly individuals, warrants careful consideration. The SOLD study evaluated the effectiveness and safety of SGLT-2i in patients aged > 70 years [232]. Although the study reported overall improvements in BMI and glycated hemoglobin with preserved renal function, a subgroup of fragile patients experienced adverse events, including urinary tract infections (UTIs) and worsening renal function, leading to treatment discontinuation. These findings underscore the need for vigilant monitoring and individualized risk-benefit assessments when prescribing SGLT-2i to elderly and fragile populations, particularly to mitigate risks such as treatment intolerance and renal function decline. SGLT-2i generally reduce body weight, which might promote sarcopenia in older individuals. In a randomized clinical trial (*N* = 129) involving elderly participants with T2DM (aged *≥* 65 years) for up to 52 weeks, EMPA treatment (10 mg once daily) did not affect muscle mass (mean difference= -0.61 kg, 95% CI -1.61–0.39) or strength ((mean difference= -0.3 kg, 95% CI -1.1–0.5) compared to the placebo group [233]. In a multicenter cohort study (*N* = 2083) involving kidney transplant recipients with diabetes, SGLT-2i was associated with a lower risk of the composite outcome of all-cause mortality, death-censored graft failure, and serum creatinine doubling than the control group that did not use SGLT-2i (adjusted HR 0.43, 95% CI 0.24–0.78; *P* = 0.006 and adjusted HR 0.45, 95% CI 0.24–0.85; *P* = 0.013, respectively) [234], suggesting that SGLT-2i may be used safely and have beneficial effects on preserving graft function in diabetic kidney transplant recipients.

## Summary and future perspectives

CKM syndrome represents a complex interplay of metabolic, renal, and cardiovascular disorders, which contribute to multi-organ dysfunction and adverse cardiovascular outcomes. A complex network of relationships exists between these diseases, highlighting the importance of considering their combined treatment. SGLT-2i may present a promising therapeutic opportunity to tackle CKM syndrome by potentially mitigating the development of underlying metabolic diseases. Alleviating glucose toxicity and renal metabolic burden represents a key mechanism to renal protection facilitated by SGLT-2i. SGLT-2i may enhance cardiac function by reducing blood pressure, influencing substrate utilization and providing additional benefits through anti-inflammatory and antioxidant effects as well as improved vascular function.

This review discussed the evidence supporting the efficacy of SGLT-2i across different stages of CKM syndrome. In CKM Stage 1, SGLT-2i may faciliate weight loss by increasing energy expenditure and inhibiting lipid synthesis genes, among other mechanisms. However, the first-line approach for patients with isolated obesity remains lifestyle intervations, including physcial activity and dietary changes. Patients with T2DM who are treated with SGLT-2i also exhibit reduction in adiposity. In CKM Stages 2–4, SGLT-2i have been shown to delay the progression of complications such as end-stage renal disease in patients with diabetes, while also reducing CV events, HHF and MACE in patients with estabilished CKD or CVD. Although SGLT-2i demonstrate well-established metabolic, renal, and cardiovascular benefits, their associated adverse effects warrant careful clinical consideration. The balance between therpapeutic benefits and risk profile depends on the individual risk status of the patient. Current clinical practice guidelines for adult patients with T2DM provide risk-stratified recommendations regarding SGLT-2i initiation, with a conditional recommendation against initiation for low-risk individuals (*≤* 3 cardiovascular risk factors without established CVD or CKD, while strongly recommending SGLT-2i initiation for high-risk patients with established CVD or CKD [200].

The management of CKM syndrome requires a comprehensive understanding of the interconnected pathophysiology involving the cardiovascular, renal, and metabolic systems. Targeting multiple organ systems through multitargeted therapies is crucial for addressing the complex interplay of cardiovascular, renal, and metabolic dysfunctions. SGLT-2i may represent a plausible future strategy, pending prospective trials in early-stage CKM populations, and may simultaneously treat multiple aspects of CKM syndrome, including improving glycemic control, preserving renal function, and reducing cardiovascular events. However, it is important to acknowledge the limitations of animal model studies in predicting human responses. Differences in physiology, metabolism, and disease progression between animals and humans may limit the direct applicability of these findings. Therefore, while animal studies provide a foundation for understanding potential mechanisms, clinical trials are essential to confirm the efficacy and safety of SGLT-2i in humans. What’s more, future research should focus on elucidating the molecular mechanisms underlying the pleiotropic effects of SGLT-2i, understanding the interconnected pathways linking the cardiovascular, renal, and metabolic systems as an integrated whole, and investigating real-world outcomes to complement findings from RCTs. Currently, there is limited research on preventive strategies for early-stage CKM syndrome. We hope to further explore the role of SGLT-2i in the early prevention of disease progression in CKM syndrome. While SGLT-2i demonstrate more pronounced effects on HF and renal outcomes, GLP-1 RAs appear to offer superior protection against atherosclerotic events, including myocardial infarction and stroke [217]. The different types of events prevented by these categories of drugs have aroused considerable interest in the potential benefits of combined treatment. Recent studies have found that given the complementary mechanism of finerenone and SGLT-2i, when used in combination, it may bring additional heart and kidney benefits [235]. For example, the CONFIDENCE trial involving patients with T2DM and CKD (*N* = 784), demonstrated that finerenone and EMPA combination therapy significantly reduced the urinary albumin-to-creatinine ratio by 29% more than finerenone alone (95% CI 0.61–0.82) and by 32% more than EMPA alone (95% CI 0.59–0.79 ), indicating its potential efficacy in renal protection [236]. Recent analyses leveraging data from large-scale randomized outcome trials of SGLT-2i (*n* = 2), GLP-1 RAs (*n* = 8), and nonsteroidal MRAs (*n* = 2) estimate that for a 50-year-old patient who initiated combination therapy, the estimated survival without major adverse cardiovascular events was 21.1 years, while that with conventional treatment was 17.9 years (an increase of 3.2 years [95% CI, 2.1–4.3]) [237]. However, a meta-analysis identified three trials in which 1743/17072 (10.2%) of T2DM patients using GLP-1 RAs received SGLT-2i at baseline. The results indicated that the effect on HHF was similarly consistent regardless of SGLT-2i use (HR 0.58, 95% CI 0.36–0.93) and HR 0.73, 95% CI 0.63–0.85; P-heterogeneity = 0.26). Effects on the composite kidney outcome (risk ratio 0.79, 95% CI 0.66–0.95) and estimated glomerular filtration rate slope (0.78 mL/min/1.73 m^2^/y, 95% CI 0.57–0.98) also did not vary according to SGLT-2i use (P-heterogeneity = 0.53 and 0.94, respectively) [238]. Therefore, clinical evidence is needed to further explore whether combination therapy is more effective than monotherapy.

## Supplementary Information

Supplementary Material 1.

## Data Availability

All data generated or analyzed during this study are included in this published article [and its supplementary information files].

## Abbreviations

AHA: American Heart Association
CKM: Cardiovascular-kidney-metabolic
CKD: Chronic kidney disease
CVD: Cardiovascular disease
OS: Oxidative stress
IR: Insulin resistance
MetS: Metabolic syndrome
T2DM: Type 2 diabetes mellitus
SGLT-2i: Sodium-glucose cotransporter-2 inhibitors
EMPA: Empagliflozin
HHF: Hospitalization for heart failure
CANA: Canagliflozin
MACE: Major adverse cardiovascular events
HFpEF: HF with preserved ejection fraction
DAPA: Dapagliflozin
NAFLD: Non-alcoholic fatty liver disease
HF: Heart failure
eGFR: Estimated glomerular filtration rate
GLP-1 RAs: Glucagon-Like Peptide-1 receptor agonists
BMI: Body mass index
ACEI: Angiotensin-converting enzyme inhibitors
RAAS: Renin-angiotensin-aldosterone system
ASCVD: Atherosclerotic CVD
PWV: Pulse wave velocity
RCTs: Randomized controlled trials
MRAs: Mineralocorticoid receptor antagonists
AMPK: Adenosine monophosphate-activated protein kinase
LPL: Lipoprotein lipase
TG: Triglycerides
VLDL: Very low-density lipoprotein
NF-κB: Nuclear factor kappa B
RECK: Reversion inducing cysteine rich protein with Kazal motifs
GLUT-2: Glucose transporter 2
DCM: Diabetic cardiomyopathy
HMGCS2: 3-hydroxy-3-methylglutaryl-CoA synthase 2
iNOS: Inducible nitric oxide synthase
ROS: Reactive oxygen species
NLRP3: NLR family pyrin domain-containing 3
HbA1c: Hemoglobin A1c
MASLD: Metabolic dysfunction-associated fatty liver disease
AKI: Acute Kidney Injury
CI-AKI: Contrast agent-induced acute kidney injury
PCI: Percutaneous coronary intervention
DKA: Diabetic ketoacidosis
