# Supplementary material for: Sweet relief: exploring mechanisms and therapeutic approaches of sodium-glucose cotransporter-2 inhibitors in cardiovascular-kidney metabolic syndrome
Source: Cardiovasc Diabetol. 2026 Apr 5;25:120. doi: 10.1186/s12933-026-03173-5 (PMC13067429; doi:10.1186/s12933-026-03173-5)
Supplement: Supplementary file 1 — Supplementary Material 1. [file 12933_2026_3173_MOESM1_ESM.docx]

**TableS1:Search strategy (from PubMed and Web of Science Core Collection )**

| Database | Search | Query |
| --- | --- | --- |
| PubMed database | #1 | SGLT-2 inhibitor |
|  | #2 | sglt2 inhibitor |
|  | #3 | Sodium-Glucose Transporter 2 Inhibitors[MeSH] |
|  | #4 | empagliflozin |
|  | #5 | dapagliflozin |
|  | #6 | canagliflozin |
|  | #7 | ertugliflozin |
|  | #8 | ipragliflozin |
|  | #9 | tofogliflozin |
|  | #10 | luseogliflozin |
|  | #11 | #1 OR #2 OR #3 OR #4 OR #5 OR #6 OR #7 OR #8 OR #9 OR #10 |
|  | #12 | Diabetes Mellitus[MeSH] |
|  | #13 | Obesity[MeSH] |
|  | #14 | Metabolic Syndrome[MeSH] |
|  | #15 | Insulin Resistance[MeSH] |
|  | #16 | #12 OR #13 OR #14 OR #15 |
|  | #17 | renal protection |
|  | #18 | Kidney Diseases[MeSH] |
|  | #19 | chronic kidney disease |
|  | #20 | Diabetic Nephropathies[MeSH] |
|  | #21 | #17 OR #18 OR #19 OR #20 |
|  | #22 | cardiorenal protection |
|  | #23 | cardiovascular protection |
|  | #24 | Cardiovascular Diseases[MeSH] |
|  | #25 | Heart Diseases[MeSH] |
|  | #26 | Heart Failure[MeSH] |
|  | #27 | #22 OR #23 OR #24 OR #25 OR #26 |
|  | #28 | Cardiovascular-kidney-metabolic |
|  | #29 | CKM syndrome |
|  | #30 | cardiorenal metabolic |
|  | #31 | #28 OR #29 OR #30 |
|  | #32 | #16 OR #21 OR #27 OR #31 |
|  | #33 | #11 AND #32 |
| Web of Science Core Collection | #1 | Diabetes Mellitus OR Obesity OR metabolic syndrome OR insulin resistance |
|  | #2 | renal protection OR chronic kidney disease OR diabetic kidney disease OR nephroprotection |
|  | #3 | cardiorenal protection OR cardiovascular protection OR cardiovascular disease OR atherosclerotic cardiovascular disease OR heart failure |
|  | #4 | Cardiovascular-kidney-metabolic OR CKM syndrome OR cardiorenal metabolic |
|  | #5 | SGLT-2 inhibitor OR sglt2 inhibitor OR empagliflozin OR dapagliflozin OR canagliflozin OR ertugliflozin OR ipragliflozin OR tofogliflozin OR luseogliflozin OR enavogliflozin |
|  | #6 | #1 OR #2 OR #3 OR #4 |
|  | #7 | #5 AND #6 |
